# Wood decay fungi show enhanced biodeterioration of low-density polyethylene in the absence of wood in culture media

**DOI:** 10.1371/journal.pone.0288133

**Published:** 2023-07-26

**Authors:** Prameesha Perera, Harshini Herath, Priyani A. Paranagama, Priyanga Wijesinghe, Renuka N. Attanayake

**Affiliations:** 1 Department of Plant and Molecular Biology, University of Kelaniya, Kelaniya, Sri Lanka; 2 Department of Chemistry, University of Kelaniya, Kelaniya, Sri Lanka; 3 Department of Botany, University of Peradeniya, Peradeniya, Sri Lanka; Aarhus University, DENMARK

## Abstract

The involvement of microorganisms in low-density polyethylene (LDPE) degradation is widely studied across the globe. Even though soil, landfills, and garbage dumps are reported to be promising niches for such organisms, recently the involvement of wood decay fungi in polyethylene degradation is highlighted. In light of this, 50 fungal samples isolated from decaying hardwoods were assessed for their wood degradation ability and for their depolymerization enzymatic activities. For the LDPE deterioration assay, 22 fungal isolates having wood decay ability and de-polymerization enzymatic activities were selected. Fungal cultures with LDPE sheets (2 cm x 10 cm x 37.5 μm) were incubated in the presence and in the absence of wood as the carbon source (C) for 45 days. Degradation was measured by weight loss, changes in tensile properties, reduction in contact angle, changes of functional groups in Fourier-transform infrared spectroscopy, Scanning electron microscopic imaging, and CO_2_ evolution by strum test. Among the isolates incubated in the absence of wood, *Phlebiopsis flavidoalba* out-performed the other fungal species showing the highest percentage of weight reduction (23.68 ± 0.34%), and the lowest contact angle (64.28° ± 5.01). Biodegradation of LDPE by *P*. *flavidoalba* was further supported by 46.79 ± 0.67% of the mass loss, and 3.07 ± 0.13% of CO_2_ emission (mg/L) in the strum test. The most striking feature of the experiment was that all the isolates showed elevated degradation of LDPE in the absence of wood than that in the presence of wood. It is clear that in the absence of a preferred C source, wood decay fungi thrive to utilize any available C source (LDPE in this case) showing the metabolic adaptability of fungi to survive under stressful conditions. A potential mechanism for LDPE degradation is also proposed.

## Introduction

Accumulation of plastic waste is a severe global concern with no viable solution. However, non-biological or biological degradation of polyethylene can mitigate this problem to some extent. Microbial biodegradation of plastics is a promising strategy to depolymerize petroleum-based plastics into monomers or mineralize them into carbon dioxide, and water [1]. Biodegradation involves biodeterioration, bio-fragmentation, bio-assimilation, and mineralization that can happen in a sequential or non-sequential manner [2,3]. In this manuscript, the term “deterioration” was used whenever native properties of polyethylene have been changed and the term “degradation” was used when the CO_2_ emission was detected during the incubation period.

By 2020, nearly 436 species of microorganisms have been reported to degrade polyethylene [2]. Most of the plastic degraders have been isolated from soils (27.8%), plastic waste dumping sites (9.6%), and composts (5.3%), while a considerable proportion was obtained from culture collections of microorganisms (15.9%). Out of these organisms, fungi such as *Fusarium oxysporum*, *Fusarium falciform*, *Purpureocillum lilacinum* [4] *Aspergillus* spp. [5], *Phanerochaete chrysosporium*, and *Trametes versicolor* [6] are some of the well-studied polyethylene degraders or deteriorators. Though the exact mode of action and the mechanism of degradation is not known, there are ample pieces of evidence of the direct involvement of white rot fungi or lignin degraders in polyethylene degradation [1,7–11]. White rot-mediated polyethylene biodeterioration could be either mechanical or enzymatic. Iiyoshi et al. [6] reported that the ligninolytic activity of white rot fungi is directly related to polyethylene degradation and particularly the involvement of manganese peroxidases in the degradation process.

Sri Lanka is a biodiversity hotspot, and it harbors tropical dry mixed evergreen forest cover as the predominant forest type. These forests are rich in economically important, decay-resistant hardwood-bearing native plant species such as *Diospyros ebenum* (Ceylon ebony/black wood), *Azadirachta indica* (Neem), and *Manilkara hexandra* (ironwood). With the persistent dry weather and decay-resistant nature, fungi, particularly white rot fungi, associated with these hardwoods should be equipped with strong and versatile ligninolytic enzyme regimes. Since white rot fungi have the ability to deteriorate polyethylene, it was hypothesized that those associated with decay-resistant hardwood-bearing native plant species are promising polyethylene deteriorators. The objectives of the present study were to isolate, and identify wood decay fungi associated with selected decay-resistant hardwood species from a tropical dry zone forest reserve in Sri Lanka, to determine their abilities to degrade wood and ligninolytic enzyme production, to evaluate their polyethylene deterioration abilities *in vitro*, to determine whether the deterioration is enhanced when no organic carbon (C) is added to the media and to propose a possible mechanism of polyethylene degradation by wood decay fungi.

## Materials and methods

### Sample selection

Thirty decayed hardwood pieces of 3–5 cm showing mainly white rot symptoms were collected in October 2020 from Dimbulagala dry zone forest reserve in Sri Lanka after obtaining the necessary approvals. Whenever possible, the plant species of the decaying hardwoods were identified, and the GPS coordinates of the locations were also recorded. Fungi were isolated from the decaying wood strips using potato dextrose agar (PDA) amended with streptomycin (100 μg/mL) and the fungicide, carbendazim (4 μg/mL) [12]. Wood pieces were surface sterilized, and 5 mm x 5 mm pieces were plated. Every colony that appeared was isolated into pure cultures and maintained in PDA. Further, 10 isolates we obtained and identified in 2018 from decaying hardwoods of the same forest reserve [12] were also used for the current study (Table 1). These 10 isolates were selected based on their variable lignin degradation abilities, ability to use wood as the C source, and the preliminary assays on polyethylene degradation. In addition, an isolate (*Curvularia lunata*) that could not effectively grow in wood-amended media in preliminary tests was also used for comparison purposes to capture the broad range of activities.

**Table 1:** Isolate names, their species identity, and vouchered/published GenBank accessions showed the highest percent similarity, the host plant species that the isolate originated.

Isolate	Species (GenBank accession)	% similarity	GenBank accession showed the highest similarity	Host plant species
DD1	*Flavodon flavus* (MZ317625)	99.53%	LC427029.1	*Diospyros quaesita*
DD2	*Phanerochaete* sp.	98.28%	LC363490.1	*Pterospermum suberifolium*
DD3	*Nigrospora oryzae* (MZ317626)	99.22%	KX985958.1	*Drypetes sepiaria*
DD5	*Hypoxylon* sp.	96.77%	FN252428.1	*Vitex pinnata*
DD6	*Nigrospora oryzae (*MZ317627)	99.60%	KX985958.1	*V*. *pinnata*
DD7	*Hypoxylon* sp.	96.91%	FM209449.1	*V*. *pinnata*
DD8	*Hypoxylon* sp.	96.91%	FM209449.1	*V*. *pinnata*
DD9	*Nigrospora oryzae* (MZ317628)	99.39%	KX985958.1	Unknown
DD10	*Curvularia lunata* (ON406154)	99.81%	MN598894.1	Unknown
DD11	*Curvularia lunata* (MZ317629)	99.80%	JN116704.1	Unknown
DD12	*Hypoxylon* sp.	96.91%	FM209449.1	*Crateva adansonii*
DD13	*Flavodon flavus* (MZ317630)	97.93%	AB971171.1	*C*. *adansonii*
DD14	*Hypoxylon* sp.	96.51%	FN252428.1	Unknown
DD15	*Coprinellus bipellis* (MZ317631)	96.87%	MT340078.1	Unknown
DD16	*Coprinellus bipellis* (MZ317632)	97.14%	MT340078.1	Unknown
DD17	*Perenniporia tephropora* (MZ317633)	99.51%	JN048763.1	*Diospyros ebenum*
DD18	*Perenniporia tephropora* (MZ317634)	99.50%	JN048763.1	*D*. *ebenum*
DD19	*Coprinellus aureogranulatus* (MZ317635)	99.54%	MH379152.1	Unknown
DD20	*Curvularia lunata* (ON406155)	99.81%	MN598894.1	*Dialium ovoideum*
DD21	*Arcopilus aureus* (ON406156)	98.29%	MH857277.1	*P*.*suberifolium*
DD22	*Paecilomyces formosus* (MZ317636)	99.82%	FJ389927.1	Unknown
DD23	*Flavodon ambrosius* (MZ317637)	97.06%	KR119074.1	Unknown
DD24	*Hypoxylon* sp.			Unknown
DD25	*Hypoxylon* sp.	96.91%	FM209449.1	Unknown
DD26	*Coprinellus bipellis* (ON406157)	96.84%	MT340078.1	Unknown
DD27	*Xylaria* sp.*			Unknown
DD28	*Xylaria feejeensis* (MZ317638)	99.62%	GU322452.1	Unknown
DD29	*Coprinellus aureogranulatus* (MZ317639)	99.69%	MH379152.1	Unknown
DD30	*Phanerochaete pseudomagnoliae* (MZ317640)	98.86%	KP135091.1	*Mimusops elengi*
DD31	*Phlebiopsis crassa*	97.50%	MT561714	*Manilkara hexandra*
DD32	*Phlebiopsis* sp.	98.05%	MT452527.1	Unknown
DD33	*Perenniporia tephropora* (MZ317641)	99.51%	JN048763.1	Unknown
DD35	*Hypoxylon fendleri* (MZ317642)	98.49%	KY173350.1	Unknown
DD36	*Hypoxylon fendleri* (MZ317643)	98.11%	KY173350.1	Unknown
DD37	*Xylaria* sp.*			Unknown
DD38	*Hypoxylon* sp.	97.04%	FM209449.1	*Bauhinia racemosa*
DD39	*Albonectria rigidiuscula* (ON406158)	98%	FN667579.1	*P*. *suberifolium*
DD40	*Coprinellus aureogranulatus* (ON406159*)*	99.69%	MH379152.1	*P*. *suberifolium*
DBP	Phanerodontia chrysosporium** (MZ087900)	99%	MH854905.1	Unknown
KH2_1	Phlebiopsis flavidoalba** (MZ087901)	99%	MT386377.1	*Aazadirachta indica*
DDW10A_1	Fusarium pseudensiforme** (MZ087902)	99%	MH863652.1	Unknown
DPW_1	Schizophyllum commune**(MZ087903)	99%	MH857808.1	Unknown
DLP_1	Schizophyllum commune** (MZ087904)	99%	MH857808.1	Unknown
DZFK2_1	Fusarium pseudensiforme** (MZ087906)	99%	MH863652.1	Unknown
DHF5B_1	Fusarium decemcellulare** (MZ087907)	99%	FN667579.1	Unknown
WDF 08	*Phanerochaete pseudomagnoliae* **(MZ087909)	98%	KP135091.1	Unknown
UKC	Aspergillus fischeri** (MZ087910)	98.90%	MH856769.1	Unknown
WDF7	*Curvularia lunata* ** (MZ317644.1)	99.22%	KP689195.1	Unknown

* Morphologically identified isolates,

** Isolates taken from the previous study [12].

### Species identification

Fungal DNA was isolated from each isolate using the CTAB protocol as described in Perera et al. [12] and stored at −20 °C until used. Polymerase Chain Reaction (PCR) was conducted with universal ITS 1 and 4 primers [13] to amplify the fungal rDNA-ITS regions. The fungal ITS region was used in species identification since it has the highest probability of tentative species identification compared to the other barcoding regions [14], and the multilocus sequence typing was not the main interest of the study. The polymerase chain reaction (PCR) mixture consisted of 1x Colorless GoTaq^®^ Flexi Buffer, 2 mM MgCl_2_, 10 mM each dNTP, 0.5 μM forward and reverse primers, 1.25 U of GoTaq^®^ DNA polymerase (Promega Inc., Madison, WI, USA), and < 0.5 μg template DNA. The PCR program was as follows: initial denaturation at 95 °C for 4 min, 40 cycles of denaturation at 94 °C for 30 seconds, annealing at 55 °C for 30 seconds, extension at 72 °C for 30 seconds, and final extension at 72 °C for 10 min (Veriti^®^ 96 Well Thermal Cycler, ABI Biosystems, Foster City, CA, USA). Whenever PCR amplification was not successful, slight variations were done to the above parameters and other universal primer pairs of the ITS region were also tried. Pure PCR products were sequenced following Sanger dideoxy chain termination technology at Genentech, Sri Lanka. Sequences were manually edited using BioEdit sequence alignment editor (Version 7.2). Using the Basic Local Alignment Search Tool (BLASTn), nucleotide sequences were compared with the authentic sequences (vouchered or published) available in the National Center for Biotechnology Information (NCBI) database, and the fungal species were identified. Sequences were deposited in the GenBank and the accession numbers were obtained (Table 1). Morphological characterization of isolates was also performed in support of species identification.

### Wood degradation abilities of fungi associated with decaying hardwood

The ability to degrade wood by the isolates was assessed by determining the percent weight loss of the wood after incubating it with fungal isolates. Forty isolates were obtained from the current study. Further, 10 previously identified species [12] were also used making a total of 50 fungal isolates. All the isolates were grown on PDA media to obtain actively growing mycelia. The wood degradation ability of all the isolates was quantified by using a modified experimental setup from Swe [15]. Briefly, a 2 mL microfuge tube was filled with 1 mL liquid medium consisting 5% (v/v) stock salt solution [NaNO_3_ (6 g/L) KCl (0.52 g/L), MgSO_4_.7H_2_O (0.52 g/L), KH_2_PO_4_ (0.82 g/L)], 0.02% (v/v) Hutner’s trace element solution [16], 0.05% (w/v) glucose, and two strips of 0.5 cm x 2 cm *Swietenia macrophylla* (Mahogany) with a weight of 0.0250 ± 0.001 g. The medium was inoculated with three mycelial discs (0.5 cm diameter) obtained from 7–10 days old actively growing colonies. Control treatments had no fungal inoculum and there were three replicates for each treatment. After a four-month incubation period, wood strips were dried in an oven at 60 °C until a constant weight is obtained, and the percent weight loss was determined. At the end of the experiment, controls remained intact without microbial contaminations. The whole experiment was repeated once.

### Low-density polyethylene (LDPE) deterioration assay

**Fig 1:**
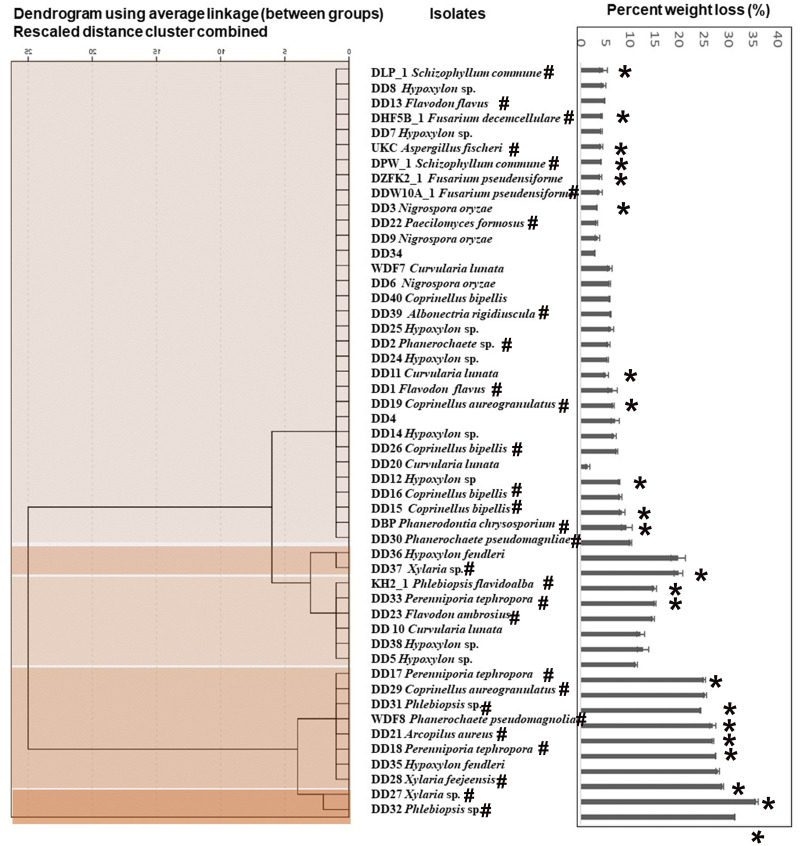
Decaying wood-associated fungal isolates showing clustering based on the variation in percent weight loss. Darker the color intensity, the higher the weight loss. Isolates used for LDPE degradation were shown with an asterisk. Isolates having lignin degradation abilities were shown in a # mark.

LDPE deterioration assay was conducted in the presence of lignocellulose and in the absence of lignocellulose. Twenty-two isolates were used for each assay. These isolates were selected based on the results of the wood decay assay conducted above (shown in Fig 1). As per results, isolates were tentatively grouped based on the wood weight loss data in categories as low, medium and high. Isolates were selected from each category, so that diverse abilities of de-polymerization enzyme production are represented in the subsequent assays. All the experiments were conducted under aseptic conditions and extra care was taken not to contaminate the flasks to make sure that the deterioration was indeed initiated by the fungus of interest.

#### LDPE deterioration in the absence of wood

Each 250 mL flask was filled with a 100 mL liquid medium consisting of 0.02% (v/v) Hutner’s trace element solution [16], and 5% (v/v) stock salt solution used for wood degradation assay described above. Transparent 37.5 μm LDPE sheets were purchased from a local polyethylene vender (Southern Lions Poly Print Pvt., Ltd., Sri Lanka). As per the producer of LDPE sheets, no specific additives were used during the production procedure using pellets (Personal communication, Southern Lions Poly Print Pvt., Ltd., Sri Lanka). Polyethylene sheets were surface sterilized with 70% ethanol for 2 min. followed by three serial washings with sterilized distilled water. A preliminary study confirmed that surface sterilization with 70% ethanol was sufficient for the complete removal of microbial contaminants in LDPE sheets [12]. Surface sterilized three LDPE sheets (2 cm x 10 cm x 37.5 μm) were added to each flask. The medium was inoculated with five mycelial discs (0.5 cm diameter) obtained from 7 to 10-day-old actively growing edges of each mycelial plate. One negative control setup contained just the medium, and another setup contained the medium with LDPE (without fungal inoculum and wood supplement). Each treatment had three replicates.

#### LDPE deterioration in the presence of wood

To determine whether lignocellulose supplementation to the media containing fungi would enhance the LDPE degradation, a separate experimental setup was arranged as indicated above. In addition, a 2 g of approximately 0.5 cm x 2 cm size *Swietenia macrophylla* (Mahogany) wood strips were supplemented to each flask. *Swietenia macrophylla*, a member of the family Fabaceae, was selected for its durability and availability in a commercial scale. Each flask was inoculated with each fungal isolate and there were three replicates per treatment. Negative control had everything but fungi in one case and in another case, only wood was added to the media in the absence of both the fungus and LDPE. Another control was maintained with wood, and LDPE in the absence of the fungus. Each treatment had three replicates and the whole experiment was repeated once more. After the incubation period of 45 days at room temperature (28–30 °C), LDPE sheets were soaked in water for 1–2 hours, carefully wiped off the mycelial mats attached to the LDPE sheets with a cotton ball while holding it under a slowly running tap water and washed with 70% ethanol followed by three serial washings with sterilized distilled water. Finally, LDPE sheets were air-dried as described previously [12]. These LDPE sheets in both experiments were subjected to the following tests to determine the changes in physical and chemical properties as described before [12].

### Changes in physical properties

Changes in physical and chemical properties were determined for all the treatments in both the experiments conducted in the presence and absence of wood as the C source.

### Percentage weight loss

After a 45-day incubation period, the percent reduction in weight of LDPE sheets after treatment was determined using the following equation [17].


$$Weight\;loss\;\left(\%\right)=\frac{\left(Initial\;weight-final\;weight\right)}{Initial\;weight}\;x\;100$$

### Reduction in tensile properties

The mechanical properties such as percentage loss of maximum tensile stress and tensile stress at yield were determined using a universal testing machine (UTM) (Instron 3365 Tensile Tester, MA, USA) available at the Sri Lanka Institute of Nanotechnology (SLINTEC).

### Changes in hydrophobicity

The wettability of LDPE was determined by the sessile drop method [10] using 15 μL of deionized water as the wetting agent. Contact angle (CA) between water and LDPE was measured with a drop shape analyzer (KRUSS DSA25, USA) available at SLINTEC, Sri Lanka. All the values were compared with the control.

### Changes in chemical properties

Fourier transform infrared (FTIR) spectra of all strips were recorded (PerkinElmer FTIR C107062, Waltham, MA, USA) using the attenuated total reflection (ATR) at the University of Kelaniya.

### Scanning electron microscopy (SEM)

Scanning electron microscopy was performed with a Hitachi SU6600 Analytical Variable Pressure FE-SEM (Tokyo, Japan) at SLINTEC for all samples as described before [12].

### LDPE degradation kinetics and biodegradability test

*Phlebiopsis flavidoalba* was isolated from a native plant species, Neem, and it showed promising results in biodeterioration in the present study as well as in our previous study [12]. Therefore, it was subjected to the strum test in the presence of polyethylene as the sole C source. The amount of CO_2_ released by *P*. *flavidoalba* was determined following the strum test according to the ISO 14855 method at Bureau Veritas, Sri Lanka. The setup consisted of treatment and, positive and negative controls. Treatment consisted of three replicate vessels containing 1 L of the salt medium, 3 LDPE strips (2 cm x 10 cm x 37.5 microns), and five mycelial disks (0.5 cm diameter) of actively growing edges of *P*. *flavidoalba* colony. Broth without the fungus was used as the blank or negative control. Further, crystalline micro cellulose was used as the reference material or positive control, and the experiment was set up in triplicates. The released CO_2_ level was measured till 70% biodegradation of the reference material was achieved. The percentage of biodegradation was calculated according to the equation described by [18]

$$Biodegradation\;\left(\%\right)=\frac{(CO2)test-\left(CO2\right)blank}{(CO2)theoretical}\;x\;100$$

where *(CO*_*2*_*) test* is the mean cumulative amount of CO_2_ produced from polymer degradation, *(CO*_*2*_*) blank* is the amount of CO_2_ released in the blank experiment, and *(CO*_*2*_*) theoretical* is the total amount of CO_2_ that would be liberated by the complete mineralization of the LDPE. The rate of CO_2_ emission was also determined.

The rate of biodegradation in the salt medium in terms of loss of mass after a 45-day incubation period was determined using the following equation [18].

$$Rd=\frac{dm}{dt}$$

where, the polymer degradation rate, *R*_*d*_, is the differential mass loss per unit time, *dm* is the mass loss, and *dt* is time.

### Changes of pH values in the media

Since the variation of pH in a culture media directly infers the metabolic activity of microorganisms, pH changes in the media were measured. The pH of the LDPE degrading medium inoculated with selected fungal species was measured before and after a 45-day incubation period using a pH meter (Consort C861, Belgium). The pH of the negative control was also determined.

### Lignin degradation and major ligninase enzyme activities

Firstly, lignin powder (Lignin alkali, Sigma-Aldrich,) amended malt extract agar (ME) medium was used to determine if the selected isolates can degrade lignin in pure cultures. Mycelial discs (5 mm diameter) from actively growing edges of 7–10 days old fungal cultures from each isolate was inoculated into the ME agar plates in triplicates per isolate. Plates were incubated at room temperature for 5 days and flooded with a freshly prepared 1% aqueous solution of FeCl_3_ and K3[Fe (CN)6]. Formation of clear zones were observed in each culture plate as a qualitative indicator of lignin utilization by the fungus [19].

Activities of three ligninase enzymes; laccase, lignin peroxidase, and manganese, peroxidase were quantitatively determined in the LDPE degrading media after the 45-day incubation period soon after the removal of polyethylene sheets from the media.

### Laccase quantification

Laccase activity was determined by the ability to oxidize ABTS [2,2’-azino-bis (3-ethylbenzothiazoline-6-sulphonic acid)] as described in [10]. Reaction volume (1 mL) consisted of 0.8 mL of the extract, 0.1 mL 600 mM acetate buffer (1.306% w/v acetic acid and 5.226% w/v sodium acetate at pH 5.0), and 0.1 mL of 5 mM ABTS. One unit of enzyme activity (U/mL) was defined as the amount of enzyme required to oxidize 1 μmol ABTS per min using an extinction coefficient of Ɛ436 = 29.3 mM^–1^ cm^–1^ [20].

### Lignin peroxidase quantification

LiP activity was quantified by the ability to oxidize veratryl alcohol in 10 mM sodium tartrate buffer [0.803% (m/v) tartaric acid and 4.464% (m/v) sodium tartrate] as described in [10]. The enzyme activity was determined by monitoring the change in absorbance at λ310 nm for three minutes at 27 °C. The reaction mixture (1 mL) consisted of 0.710 mL extract, 0.2 mL tartrate buffer, 0.05 mL 8.12% (v/v) H_2_O_2_, and 0.04 mL 3.65% (v/v) veratryl alcohol. One unit of enzyme activity (U/mL) was defined as the amount of enzyme required to oxidize 1 μmol veratryl alcohol per minute using an extinction coefficient of Ɛ310 = 9.3 mM^–1^ cm^–1^ [20].

### Manganese peroxidase (MnP) activity

MnP activity was assessed by determining the oxidation of guaiacol as the substrate. The reaction mixture consisted of sodium succinate buffer (0.5 M, pH 4.5), 4 mM guaiacol, 1 mM manganese sulphate, and crude enzyme extract. The mixtures were incubated for 1 minute at 27 °C. The reaction was initiated by the addition of 6% H_2_O_2_ and the absorbance was measured at 465 nm with an extinction coefficient of Ɛ465 = 12.100 mM^-1^cm^-1^ [21]. One unit of the enzyme was determined as indicated above.

### Statistical analysis

Grouping of isolates based on percentage wood weight loss was done following the Squared Euclidean distance method. Significant differences among treatment units were determined using one-way ANOVA and Pearson correlation analysis was performed between enzyme activities in salt medium and LDPE degradation measurements using IBM SPSS version 23.

## Results

### Fungal species identification

Altogether, pure cultures of 40 fungal isolates were obtained in the present study. Additionally, 10 isolates used in our previous study [12] were also included making it a total of 50 isolates. All these isolates are maintained at the fungal culture collection of the Department of Plant and Molecular Biology, University of Kelaniya Sri Lanka. Isolates were identified up to the species levels or at least to the genus level using both morphological and ITS sequence analysis (Table 1). Two isolates that failed to identify with DNA bar code data due to a lack of high similarity values in BLASTn searches were not included in Table 1.

### Wood decay ability of the fungal isolates

As shown in Fig 1, the percent weight loss of wood ranged from 1.41% to 35.70%. Hierarchical clustering based on the percent wood weight loss of the isolates was represented by five groups (Fig 1). *Xylaria* sp. (DD 27), and *Phlebiopsis* sp. (DD32) belonged to cluster 1 showing the highest weight reduction ranging from 31% to 35%. The second cluster consisted of moderate wood degraders that ranged from 24% to 29%. All the isolates belonging to cluster three showed weight loss of approximately 19%. Six isolates clustered to form group four showed weight reduction in the range of 15% to 11%. The least wood degraders formed the fifth cluster ranging from 1% to 10% weight loss.

### Ligninase activity in LDPE containing media

**Fig 2:**
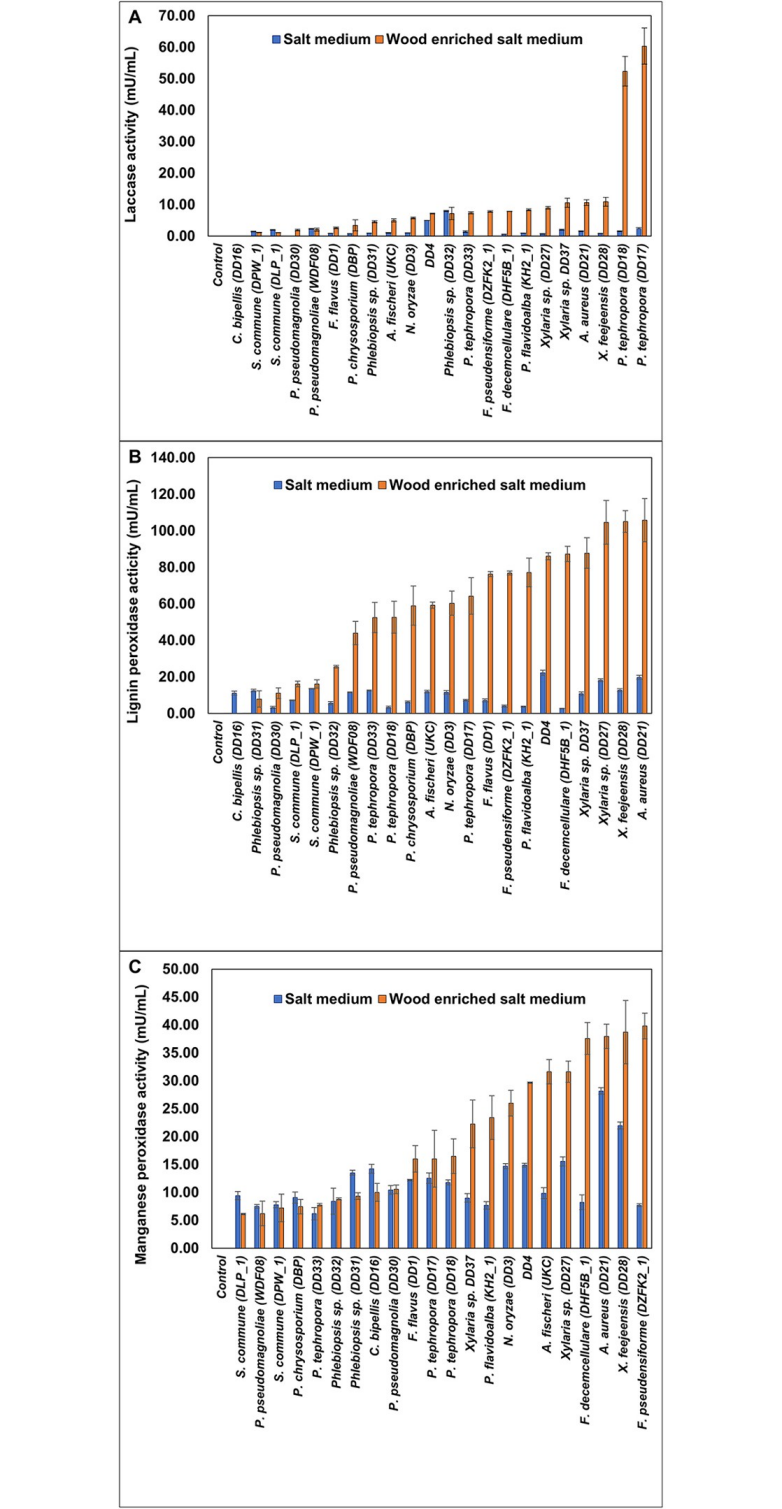
Enzyme activity of the LDPE degrading salt medium and wood-enriched salt medium after the 45-day incubation period. (A) Laccase (B) Lignin peroxidase and (C) Manganese peroxidase activities. Whiskers show one standard error (n = 3).

After the 45-day incubation period, laccase, LiP, and MnP activities in the absence of wood and in the presence of wood in the salt media were measured. A comparison of enzyme activities in each medium is illustrated in Fig 2.

In general, wood enrichment in the media elevated the activities of all three enzymes. For example, laccase production in the salt medium was in the range from 0.64 ± 0.03 mU/mL (*F*. *decemcellulare* DHF5B) to 7.95 ± 0.17 mU/mL (*Phlebiopsis* sp., DD32). In contrast, laccase activity in wood amended media ranged from 1.2 ± 0.08 mU/mL (*S*. *commune*, DPW1) to 60.34 ± 5.75 mU/mL (*P*. *tephrophora*, DD17). Orderly, 25.67 and 33.99-fold increments of laccase activity by *P*. *tephrophora* DD17 and *P*. *tephrophora* DD18 were detected when the media was supplemented with woods compared to the medium that lacks wood amendment. In both media, *C*. *bipellis* DD16 showed no laccase production.

LiP amounts in the salt medium were in the range of 2.70 ± 0.11 mU/mL (*F*. *decemcellulare* DHF5B*)* to 22.24± 1.36 mU/mL unidentified DD4 strain and wood amendment increased the activity from 7.93 ± 4.48 mU/mL (*Phlebiopsis* sp. DD31) to 105.77 ± 11.78 mU/mL (*A*. *aureus*, DD21). Interestingly, though *C*. *bipellis* DD16 showed no LiP activity in wood amended medium, elevated activity of 11.06 ± 1.09 mU/mL was detected in the salt medium in repeated experiments.

MnP activity ranged from 6.19 ± 1.13 mU/mL (*P*. *tephrophora*, DD 33) to 28.17 ± 0.58 mU/mL (*A*. *aureus*, DD21) in the salt medium and it ranged from 6.10 ± 0.11 mU/mL (*S*. *commune*, DLP_1) to 39.81 ± 2.3 mU/mL (*F*. *pseudensiforme* DZFK2) in the wood amended medium.

A strong positive correlation between MnP and LiP in both salt and wood amended salt media were observed (ρ = 0.623, p = 0.00; ρ = 0.777, p = 0.00 respectively). The striking feature here is that in the LDPE media when no other C source is given, fungi could display enzymatic activities even at a reduced level.

### Change of pH values in the media

As shown in Table 2, the initial pH was 5.4 in the control experiments (salt medium with LDPE and the salt media without LDPE) and it remained unchanged even after 45 days. After the 45-day incubation period, pH values of the treatments (inoculated with fungi in the absence of wood) showed a slight increase ranging from 6.7 to 8.2. However, the majority of the isolates maintained around a neutral pH value. After the 45-day incubation period, the pH values changed from 5.4 to 4.4 in the control experiments of wood-amended media indicating the addition of wood has caused a reduction of pH in the medium. In other words, compounds present in the wood maintained an acidic pH in the salt medium. pH values varied approximately between 3.9 to 6.3 in the treatments (inoculated with fungi) after the incubation period. Only six fungal isolates decreased pH values below 4.4 (yet in the range of 3.9 to 4.1) in the presence of wood in the media (Table 2).

**Table 2:** Mean pH change in the LDPE degradation medium inoculated with selected fungal isolates (not showing unidentified species) after the 45-day incubation period.

Number	Isolate	Salt medium (without wood)	Wood-amended salt medium
	Initial medium	5.4 ± 0.00^a^	5.4 ± 0.00
	Control	5.4 ± 0.04^a^	4.4±0.03 ^a^
1	*Flavodon flavus* (DD1)	6.7 ± 0.00	4.0±0.07 ^a^
2	*Phlebiopsis flavidoalba* (KH2_1)	6.7 ± 0.00	3.9±0.03
3	*Phanerodontia chrysosporium* (DBP)	7.2 ± 0.00	5.0±0.29
4	*Perenniporia tephropora* (DD17)	7.3 ± 0.00	4.1±0.07 ^a^
5	*Phanerochaete pseudomagnolia* (DD30)	7.3 ± 0.00	5.4±0.03
6	*Phlebiopsis* sp. (DD32)	7.3 ± 0.00	4.4±0.07 ^a^
7	*Phanerochaete pseudomagnoliae* (WDF08)	7.3 ± 0.26	4.5±0.03 ^a^
8	*Perenniporia tephropora* (DD18)	7.4 ± 0.14	4.3±0.09 ^a^
9	*Aspergillus fischeri* (UKC)	7.7 ± 0.33	6.1±0.12
10	*Arcopilus aureus* (DD21)	7.7 ± 0.35	5.2±0.26
11	*Fusarium decemcellulare* (DHF5B_1)	7.8 ± 0.32	6.1±0.03
12	*Xylaria feejeensis* (DD28)	7.8 ± 0.00	6.3±0.03
13	*Phlebiopsis* sp. (DD31)	7.8 ± 0.00	4.3±0.07 ^a^
14	*Xylaria* sp. (DD27)	7.9 ± 0.00	5.4±0.12
15	*Fusarium pseudensiforme* (DZFK2_1)	7.9 ± 0.00	6.2±0.23
16	*Xylaria* sp. DD37	7.9 ± 0.02	5.4±0.15
17	*Perenniporia tephropora* (DD33)	7.9 ± 0.06	3.9±0.06
18	*Schizophyllum commune* (DLP_1)	8.0 ± 0.00	4.6±0.03 ^a^
19	*Schizophyllum commune* (DPW_1)	8.1 ± 0.00	4.6±0.03 ^a^
20	*Nigrospora oryzae* (DD3)	8.2 ± 0.00	5.1±0.03
21	*Coprinellus bipellis* (DD16)	8.2 ± 0.00	4.8±0.12 ^a^

^a^ Significant according to Dunnett’s multiple comparisons with the control. In each column, the values that are not labeled with the letter “a” are significantly different from the mean value of the control.

The initial pH was measured at the beginning of the experiment and the final pH was measured at the end of the 45-day incubation period.

### Assessment of polyethylene degradation

#### Percentage weight loss of LDPE

**Fig 3:**
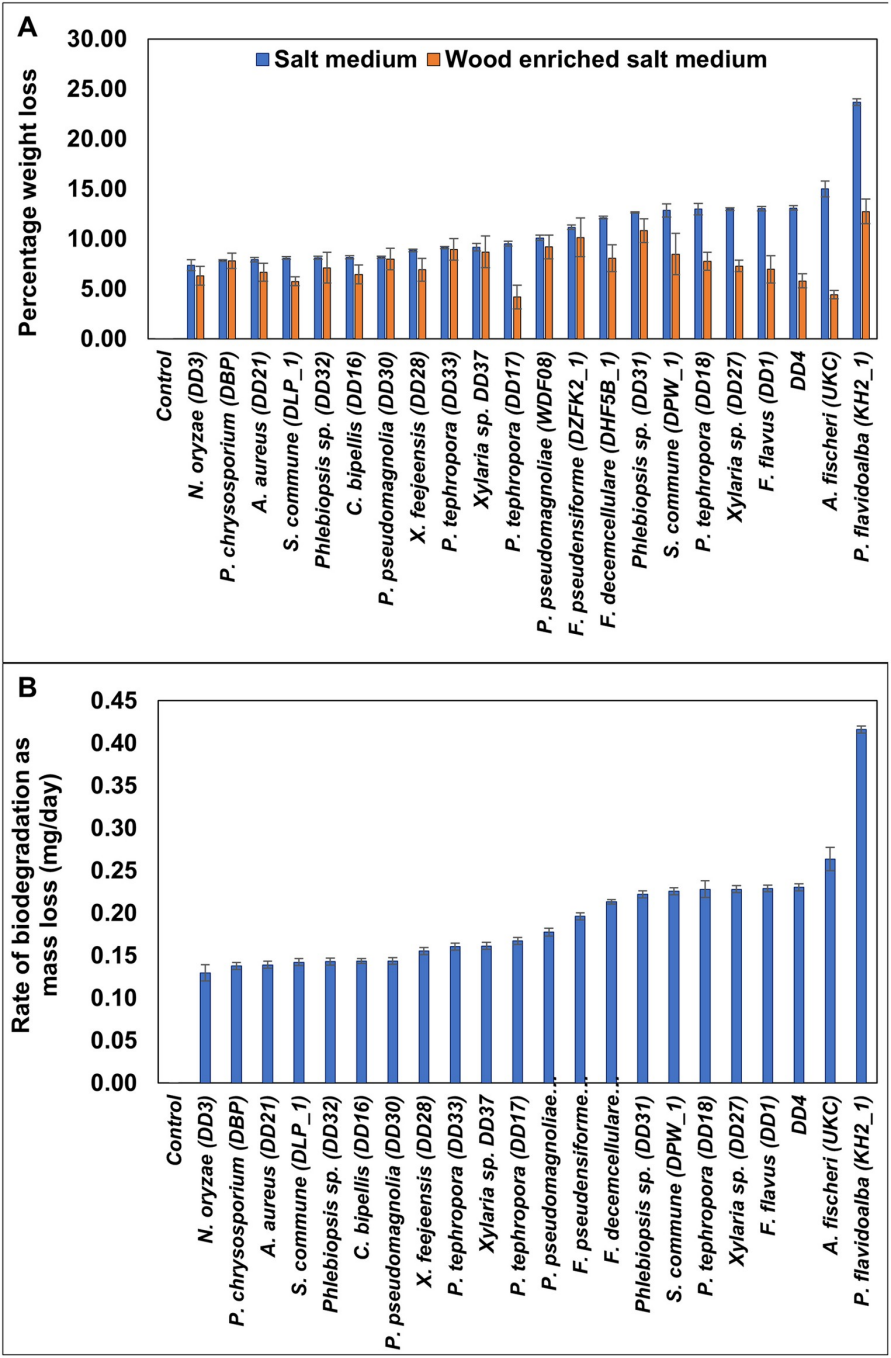
Percentage weight loss (A), and deterioration kinetics (B) of LDPE sheets in salt and wood enriched salt media after a 45-day incubation period. Whiskers show one standard error (n = 3).

Percent weight loss of LDPE after the incubation period was shown in Fig 3A. Always in all the cases, the highest percentage of LDPE weight loss was observed in the absence of wood in the media. While the percent weight loss in salt media ranged from 7.37% to 23.68%, wood-enriched media had only a 4.18% to 12.76% reduction. On both occasions, *P*. *flavidoalba* KH2 treated with LDPE strips showed the highest percentage of weight loss. A nearly two-fold increase in weight loss was observed when polyethylene enriched salt medium was inoculated with *P*. *tephrophora* DD17, *P*. *tephrophora* DD18, *Xylaria* sp. DD27, *F*. *flavous* DD1, and *P*. *flavidoalba* KH2. *Aspergillus fischeri* UKC showed a three-fold increase in percentage weight loss in the salt medium compared to that of the wood amended medium. *Phanerodontia chrysosporium* DBP showed no significant difference in weight loss in both media. As shown in Fig 3B, *P*. *flavidoalba* KH2 showed the highest biodegradation rate as inferred by mass loss per unit time (0.42 ± 0.00 mg/day) followed by *A*. *fischeri* UKC (0.26 ± 0.01 mg/day).

#### Reduction in mechanical properties of LDPE

**Fig 4:**
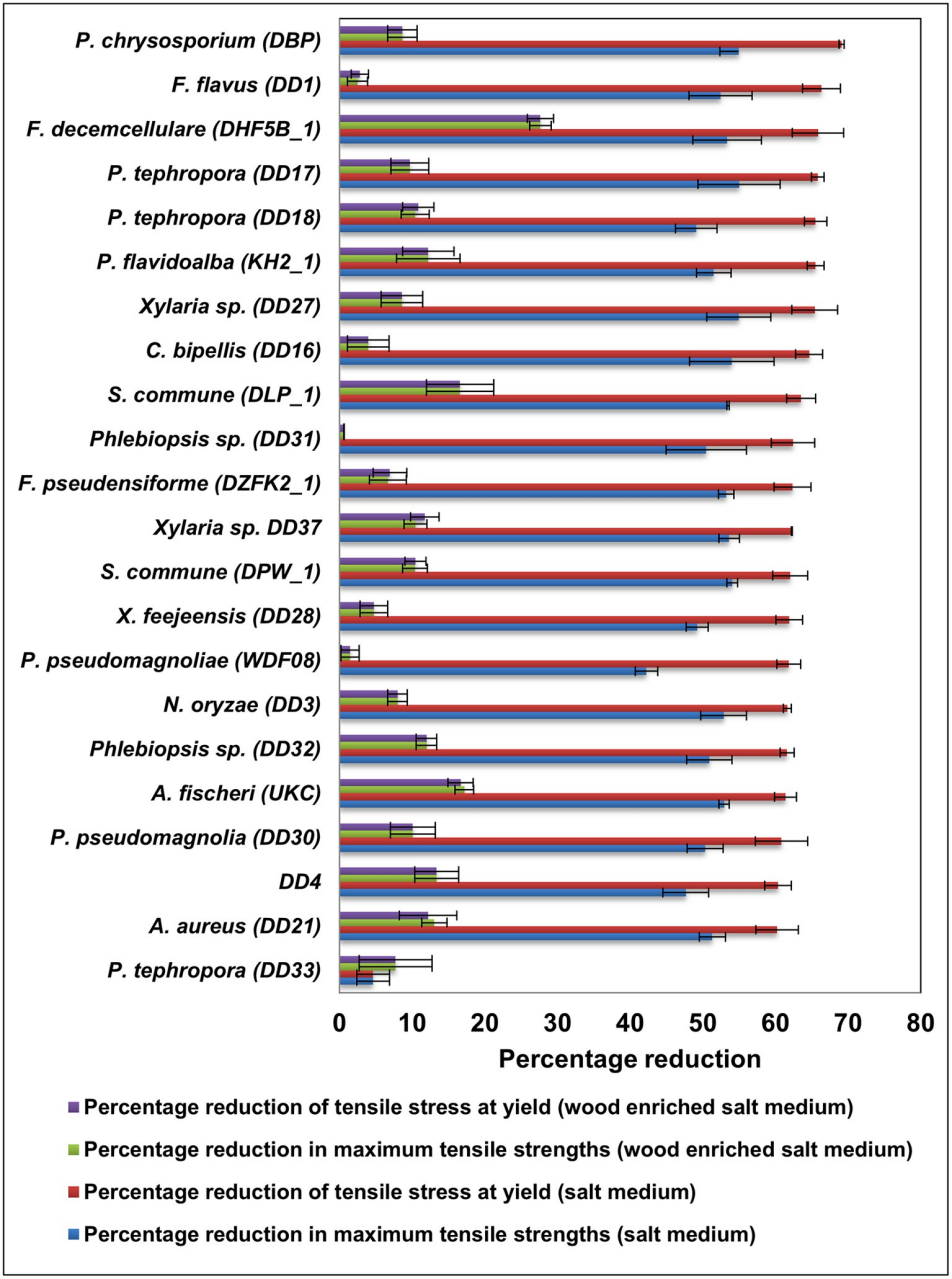
Reduction in mechanical properties; the percent reduction in maximum tensile stress and percent reduction in tensile stress at yield by each fungal isolate in each medium. Whiskers show one standard error (n = 3).

The percentage reduction in tensile properties in LDPE sheets is shown in Fig 4. Tensile properties of control remained constant while the percent reduction of tensile stress at yield was higher in the absence of woodchips in the media than that in the presence of woodchips (shown in red and purple colors in the Fig 4). Percent reduction of tensile stress at yield ranged from 4.64 ± 2.26% to 69.11 ± 0.35%. All the values reported were higher than 60% except for *P*. *tephrophora* DD33. A similar pattern was observed for the percentage reduction in maximum tensile strengths as well, which ranged from 4.64 ± 2.26% to 55. 01 ± 5.64% (shown in blue and green in the Fig 4). *Perenniporia tephrophora* DD17 treated LDPE was given the highest percent reduction in maximum tensile strength followed by *Xylaria* sp. DD27 and *P*. *chrysosporium* DBP. All the values were higher than 40% except for one *P*. *tephrophora* DD33 isolate. It was interesting to note that the percent reduction in tensile properties was less than 17% in almost all the samples in wood amended medium except for *F*. *decemcellulare* DHF5B, and *P*. *tephrophora* DD33.

#### Hydrophobicity measurement

**Fig 5:**
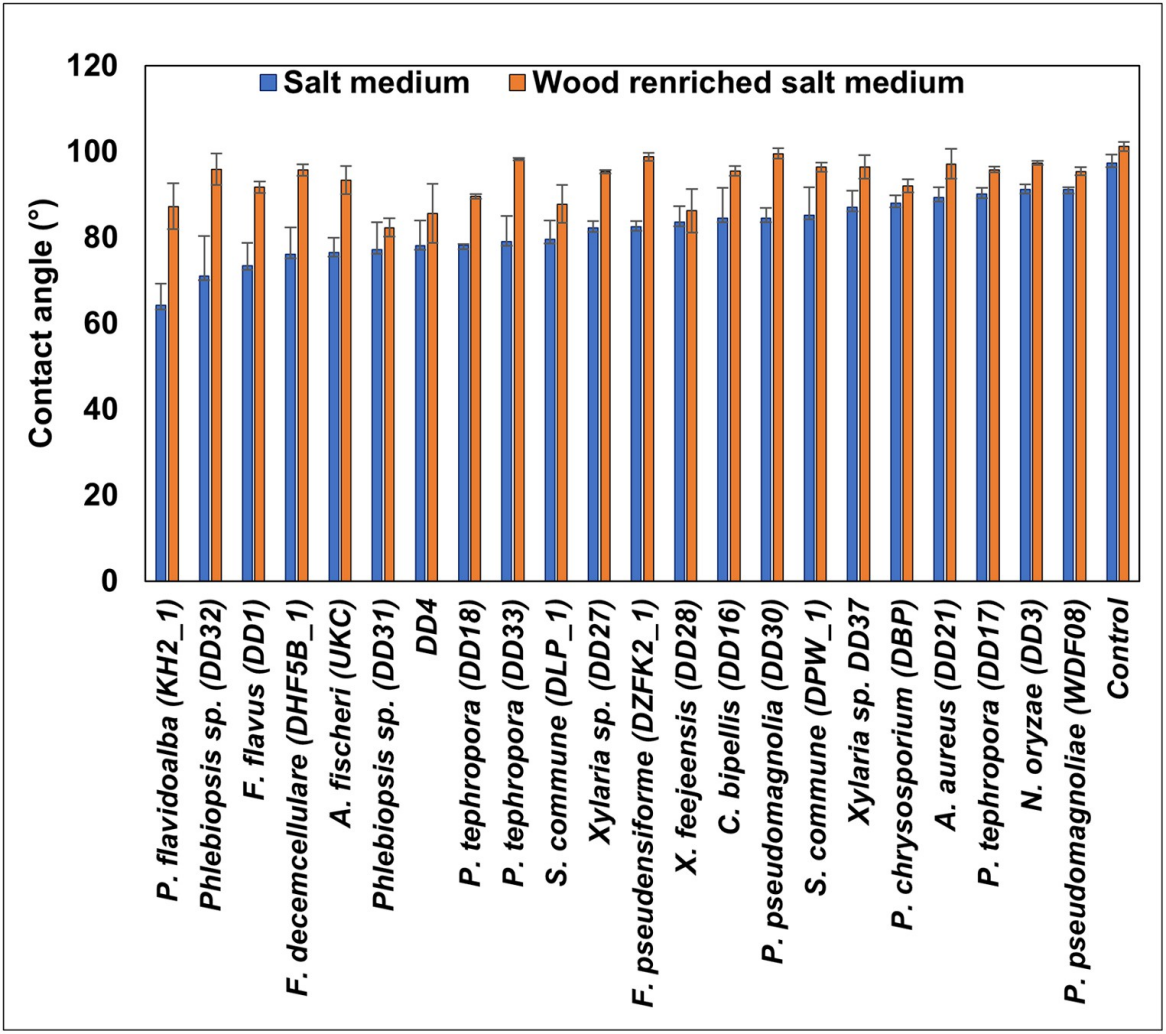
Comparison of means of contact angle of 37.5-micron LDPE deteriorated by fungal isolates after a 45-day incubation period in salt and wood amended salt medium. Whiskers show one standard error (n = 3).

Reduction in hydrophobicity of LDPE is an indication of degradation/deterioration and a contact angle (CA) less than 90° is considered a hydrophilic surface [22]. Control samples showed a CA of 97.35° ± 1.92 and 101.18° ± 1.08 in salt and wood amended salt medium respectively. In both treatments, there was a clear reduction in CA compared to the control (Fig 5). When wood is absent in the medium, *P*. *flavidoalba* KH2 showed the least CA value (64.28° ± 5.01), followed by *Phlebiopsis* sp. DDD32 with 71.03°± 9.34.

#### Fourier transformed infrared spectroscopy of LDPE

**Fig 6:**
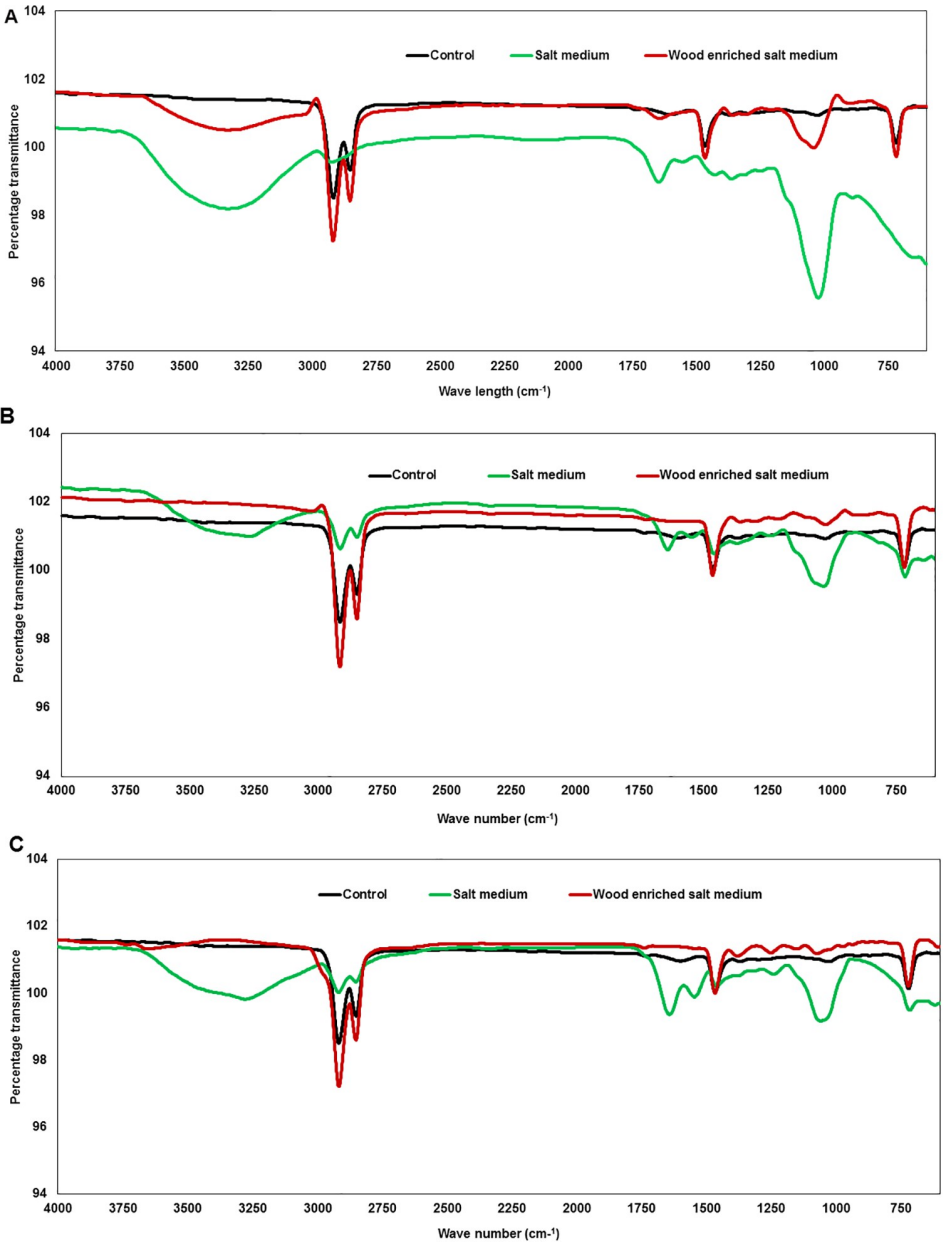
FTIR of biodegraded LDPE strips in the salt medium after 45 days of the incubation period. (A) *P*. *flavidoalba* (KH2), (B) *S*. *commune* (DLP_1), (C) *C*. *bipellis* (DD16) treated LDPE. LDPE sheets treated with *P*. *flavidoalba* showed peaks at 3200–3600 cm^-1^ indicating OH stretching. Further, peaks at 1000–1200 cm^-1^ corresponded to -C-O stretch due to carboxylic acids, esters and ethers. New peaks appeared at 1639 cm^-1^ corresponding to C = O in aldehydes/ketones and at 1297 cm^-1^ corresponding to COC of ether in LDPE sheets incubated with *P*. *flavidoalba* in wood enriched media.

FTIR analysis of LDPE was done for all 22 samples and controls after a 45-day incubation period in both treatments. The prominent peaks of LDPE at 2918 cm^-1^ and 2851 cm^-1^ in controls were attributed to asymmetric and symmetric C-H stretching (SP^3^), respectively [23]. A peak at 1466 cm^-1^ attributes to C-H bending of CH_2_, and a peak at 720 cm^-1^ attributes to = C-H bending vibration [24]. Appearance, disappearance, and peak shifts were observed in both media when incubated with the fungal isolates. Most chemical changes were prominent in the absence of woods. In salt medium, broad peaks between 3200–3600 cm^-1^ were observed due to OH stretching [25,26] in *F*. *flavus* DD1, *C*. *bipellis* DD16, *S*. *commune* DLP_1, *P*. *flavidoalba* KH2, and *A*. *fischeri* UKC while the same functional group was present even in wood enriched media treated with *Phlebiopsis* sp. DD31 and *P*. *flavidoalba* KH2. The peaks in the 1000–1200 cm^-1^ region of the FTIR spectrum correspond with -C-O stretch due to, carboxylic acids, esters and ethers [17]. This peak appeared when the LDPE in the salt medium was inoculated with *F*. *flavus* DD1, *C*. *bipellis* DD16, *Phlebiopsis* sp. DD31, *P*. *flavidoalba* KH2, and *A*. *fischeri* UKC whereas the same peak disappeared in wood amended medium inoculated with *N*. *oryzae* DD3, *C*. *bipellis* DD16, *Xylaria* sp. DD27, *X*. *freejensis* DD28, *Phlebiopsis* sp. DD32, *P*. *tephrophora* DD33, *F*. *decemcellulare* DHF5B, *S*. *commune* (DLP_1 and DPW_1), *P*. *flavidoalba* KH2, *A*. *fischeri* UKC, and *P*. *pseudomagnoliae* WDF8. Aldehydes and ketones are intermediate products of biodeterioration of LDPE. The peaks responsible for the formation of these groups also indicate biodeterioration [17]. A peak due to C = O of aldehydes/ ketones was present at 1640 cm^-1^ in deteriorated LDPE sheets by *S*. *commune* (DLP_1) in the salt medium and, *Phlebiopsis* sp. DD31, and *P*. *flavidoalba* KH2 treated wood enriched salt medium. Other than these changes functional groups for ether, esters, and alkene compounds were present in both treatments (Fig 6). It is interesting to note that the peak at 2851 cm^-1^ disappeared in *P*. *flavidoalba* (KH2)-treated LDPE in the salt medium indicating degradation of LDPE. Repeated analysis produced the same results.

#### Scanning electron microscopy of LDPE

**Fig 7:**
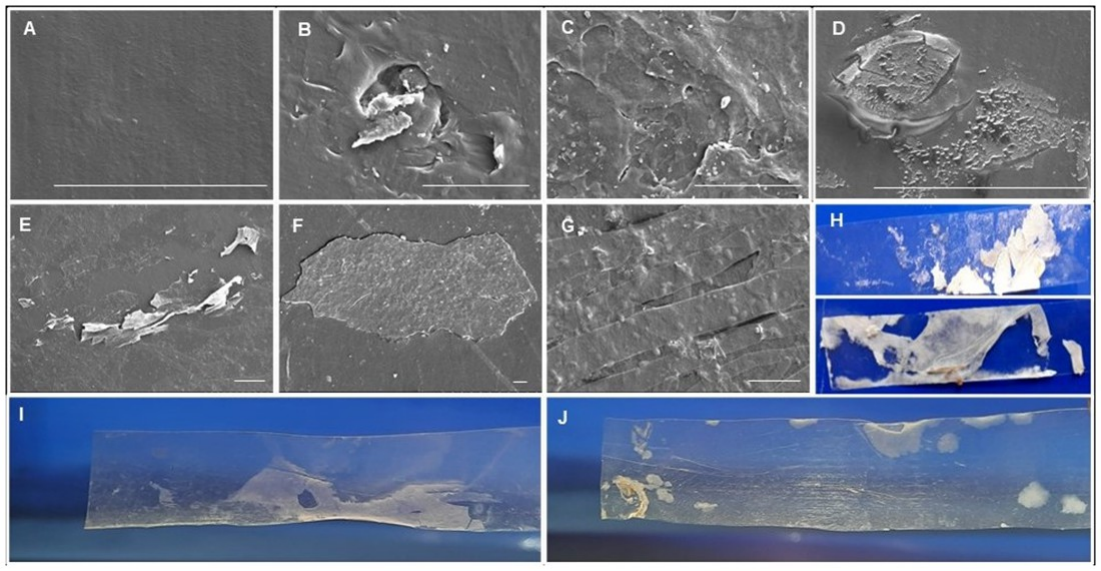
Scanning Electron Microscopy, mycelial aggregation, and distortions in degraded low-density polyethylene strips after 45 days. (A) Control, (B) *P*. *tephrophora* (DD18), (C) *Xylaria* sp. DD 27, (D*) X*. *freejensis* DD28, (E) *Phlebiopsis* sp. DD 31, (F) *P*. *flavidoalba* KH2 treated microns 37.5 stripes. (G) and (H) Mycelia attached to the LDPE (un washed LDPE sheet with mycelia), (I) to (J) macroscopic distortions of *P*. *flavidoalba* KH2 and *Xylaria* sp. DD 27 treated LDPE. Scale bars represent the 10 μm length.

SEM analyses confirmed prominent modifications on the surfaces of LDPE films and the weakening of their physical integrity due to microbial action [27]. SEM showed that the fungal-treated LDPE sheets became rough showing cracks, holes, and signs of peeling-off regions whereas the untreated sheets retained a smooth surface even after 45 days of incubation under the same conditions in both types of media (Fig 7 and S1 Fig in S1 File).

Mycelial growth on the LDPE in wood-amended salt media was higher than that of the salt medium as seen under the naked eye. However, directly observable macroscopic changes such as distortions and changes in the shape of the strips and permanent patches on the LDPE sheets were observed in salt media inoculated with *P*. *flavidoalba* KH2, *F*. *flavus* DD1, *P*. *tephrophora* DD17, and DD18, *Xylaria* sp. DD 27, DD37, *X*. *freejensis* DD28, *Phlebiopsis* sp. DD 31, and DD32, and *A*. *fischeri* UKC in the absence of wood. Control sheets remained intact during the experimental period and no contaminations were detected. Such directly observable changes were not detected in the wood-amended medium. Peeling off of the LDPE layers was also noted on the samples treated with *P*. *flavidoalba* and *Xylaria* sp. DD27. It should be noted that the samples treated with these two fungal species failed several times in SEM since the sheets were weakened and unable to face the gold sputtering process (Personal communication with the SEM service center, SLINTEC, Sri Lanka).

#### Strum test to confirm the biodegradation of LDPE by *P*. *flavidoalba*

**Fig 8:**
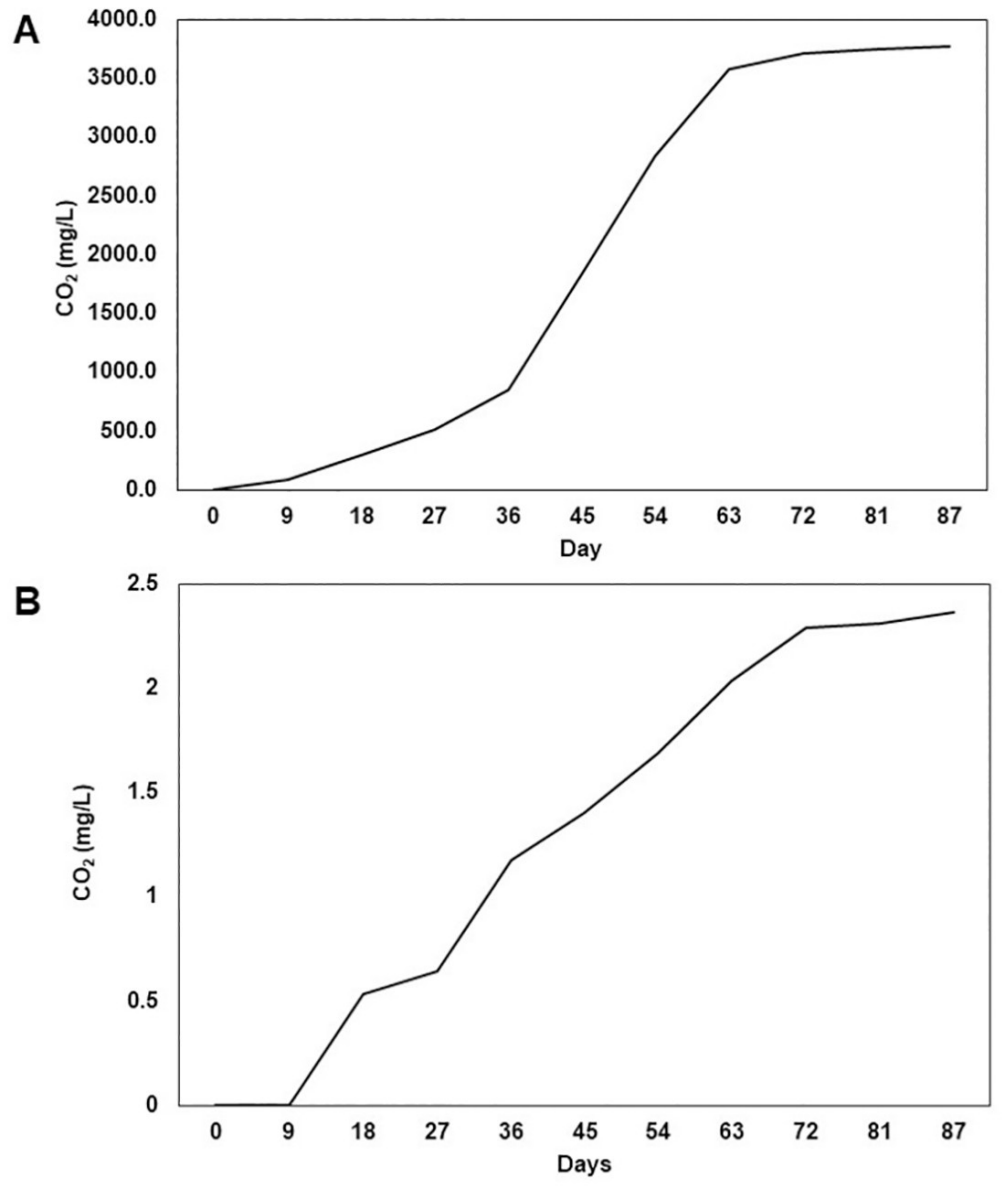
Evolution of CO_2_ by *P*. *flavidoalba* after degrading (A) Crystalline micro cellulose and (B) 37.5-micron LDPE sheets.

Mineralization of LDPE by *P*. *flavidoalba* KH2, the best deteriorator from the above experiments, was measured as the release of CO_2_ in the strum test. The level of CO_2_ released by *P*. *flavidoalba* KH2 resulted from LDPE degradation and crystalline micro cellulose degradation as the positive control is illustrated in Fig 8. Both materials showed a similar pattern/trend of degradation after 87 days. By the end of the experimental period, *P*. *flavidoalba* KH2 released 2.37 mg/L CO_2_ as a result of the mineralization of LDPE compared to the 3775.9 mg/L in the reference material, crystalline micro cellulose (Fig 8).

Biodegradation is expressed in terms of percent weight loss of LDPE sheets as well as percent release of CO_2_ compared to the control. It is evident that percent weight loss was nearly doubled from 45 to 88-day incubation period and a similar trend was observed in CO_2_ release as well (Table 3).

**Table 3:** Percentage biodeterioration and biodegradation of LDPE and the crystalline cellulose by *P*. *flavidoalba* in the salt medium calculated in terms of weight loss and release of CO_2_.

Duration	Percentage biodeterioration as mass loss of LDPE (%)	Percentage biodegradation as the release of CO_2_ (%)	
		LDPE	Reference material
After 45 days	23.68 ± 0.34	1.49 ± 0.15	29.95 ± 0.00
After 88 days	46.79 ± 0.67	3.07 ± 0.13	70.93 ± 0.00

Since the best performance of LDPE degradation was observed in the absence of wood in the media, correlation analysis was performed only for the data set obtained for the salt media (in the absence of wood supplement) assays. Significant and positive correlations (though not very strong) were observed between percentage weight loss and percent reduction in tensile strength (r = 0.418, p = 0.000). Percent weight loss was also positively and significantly correlated with a tensile strength at yield as well (r = 0.466, p = 0.000) in the absence of wood in the media. Moreover, positive correlations were obtained between percentage reduction of maximum tensile stress as well as percentage reduction of tensile stress at yield with MnP (r = 0.435, p = 0.00 and r = 0.417, p = 0.000 respectively). pH change in the media also positively correlated with the percentage reduction in maximum tensile stress (r = 0.436, p = 0.00) and the reduction of tensile stress at yield (r = 0.417, p = 0.00) indicating that pH change also has a slight influence on LDPE degradation (Table 4).

**Table 4:** Correlations among enzymes, degradation parameters, and pH in the salt medium (absence of wood).

	Laccase	LiP	MnP	% WL	%MTS	%TSY	pH
Laccase	1						
						
LiP	0.215	1					
	0.076						
MnP	-0.012	0.604**	1				
	0.92	0					
%WL	0.018	0.112	-0.021	1			
	0.886	0.358	0.867				
%MTS	0.085	0.149	0.381**	0.418**	1		
	0.488	0.223	0.001	0			
%TSY	0.11	0.133	0.359**	0.466**	0.941**	1	
	0.369	0.277	0.003	0	0		
pH	0.017	0.484**	0.328**	0.108	0.436**	0.382**	1
	0.892	0	0.006	0.379	0	0.001	

** Correlation is significant at 0.01 level (shaded), LiP: Lignin peroxidase, MnP: Manganese peroxidase, %WL: Percentage weight loss, %MTS: Percentage reduction in maximum tensile strength, %TSY: Percentage reduction in tensile strength at yield.

## Discussion

Being heterotrophic organisms, fungi are well adapted to degrade complex materials using an array of versatile enzymes and they often display metabolic flexibilities [28]. In light of this, we tested the LDPE deterioration abilities of selected hardwood decay fungal isolates in Sri Lanka. What is unique about these isolates is that, most of the fungal isolates were originated from decay-resistant hardwood-bearing plant species that are native to Sri Lanka. For an example, the white-rot fungus *F*. *flavus* was isolated from super luxury class wood *Diospyros quaesita* (Calamander) wood, which is endemic to Sri Lanka and categorized as endangered in the IUCN Red List of Threatened Species in 2020 (the national red list, 2020). Two *P*. *tephrophora* isolates were found from decaying woods of the *Diospyros ebenum* plant, which is another vulnerable species producing extremely durable jet black timber. Though it was expected to detect better deterioration of LDPE in the presence of wood in the media since wood chips induce the production of strong depolymerizing enzymes, a higher level of deterioration was observed in the absence of wood in the media. Several lines of evidence supported this claim.

The first line of evidence comes from the weight loss data of LDPE. It is the simplest and most direct way of quantifying the extent of degradation [17,18]. Partial conversion to small molecules (including CO_2_ and H_2_O) and their resulting volatilization or solubilization leads to the reduction in the mass of non-volatile or insoluble polymeric material [29]. However, the overall mass loss convolutes the liberation of small molecules with the flaking of larger, insoluble pieces, including microplastics (0.5–5 mm) and mesoplastics (5−200 mm) [30]. Though in some cases there was a high SD in weight loss most likely due to instrumental issues, the general trend in weight reduction in both media gives a strong evidence that the weight reduction is not just an artifact. Further, the percent weight loss of LDPE was higher in the absence of wood than in the presence of wood in the media all the time. This difference was nearly a two-fold increase in some instances (*P*. *flavidoalba* KH2, *A*. *fischeri* UKC, and *P*. *tephrophora*- DD17). We previously reported that the 20-micron LDPE degradation ability of *P*. *flavidoalba* KH2, *S*. *commune* DLP_1, and DPW_1, and *P*. *chrysosporium* DBP with a percentage weight loss of 2.6%, 9.65%, and 2.5%, respectively when incubated in Czapek-Dox broth after 45 days [12]. In this study 37.5-micron LDPE sheets were used since in our preliminary survey it was found that 37.5-micron is the most commonly used PE type. After 45 days of incubation, the same isolate had higher percent weight loss than that of the Czapek-Dox broth media [12].

The second line of evidence of enhanced deterioration in the absence of wood in the media came from the reduction of tensile properties. It is reported that LDPE films become fragile when it was cultured with fungal species [12,31]. In the present study, all the tested fungal isolates showed elevated percent reduction in tensile stress at yield and in maximum tensile strength. As an example, the percent reduction in maximum tensile stress of LDPE treated with *P*. *flavidoalba* KH2 in salt media was 5.4 times higher than that of the wood amendment.

Thirdly, striking changes in the FTIR spectrum was observed in the absence of wood in the media. Modifications of the FTIR spectrum reflected the change in carbon backbone and helped understanding the formation or disappearance of functional groups on LDPE or any molecule formed during the deterioration process. This technique has been widely utilized to display the chemical changes during plastic biodegradation [27,32,33]. In the present study also, we observed the changes in bond scission, chemical transformation, formation, and disappearance of new functional groups in the LDPE films. Degradation has been explained by fungal enzymatic activities. For example, it is reported that laccase-mediated oxidative cleavage of the amorphous region of polyethylene films results in the formation of easily accessible carbonyl groups [34]. The appearance of the peaks at 3264–3421 cm^-1^ (broadband) corresponds to the O-H vibration of acids [35]. These changes could be attributed to the presence of organic carboxylic acid produced from hydrolysis reactions by fungal enzymes. Bands at 700–900 cm^-1^ corresponding to -C = C- stretching and the presence of alkene groups found in the LDPE also indicated the hydrolysis reactions most likely by fungal activities [36]. But should be noted that internal defects in the polymers might have facilitated the enzyme binding abilities through weak points might that have sped up the degradation and show relevant FTIR peaks. However, in all the cases we assayed using 20-plus isolates, similar trends were observed and therefore, simply polyethylene defects may not have been the sole reason for deterioration.

The fourth line of evidence comes from the contact angle measurements. The ability of microorganisms to utilize any substrate depends on their growth and adherence to that substrate. It is believed that the more hydrophilic the surface is easier it is to be colonized by microorganisms [18,37]. Here also report that the reduction in contact angle (CA) was higher under C-limited culture conditions than that of C-enriched cultures by the end of incubation period. In support of this, Santo et al. (2013) [38] demonstrated that the microbial cell surface becomes more adhesive in carbon-starved cultures than in non-starved cultures. In the present study, all 22 isolates showed reduced CA when the media is not enriched with C. In hydrophilic surfaces, with high wettability results in lower contact angles [10,18]. This is attributed to polar group formation, such as carbonyl (C = O), carboxyl (O = C-OH), and hydroxyl (-OH) [39], which was confirmed by the FTIR analysis in the present study.

Though not quantified, SEM data also support the evidence of enhanced degradation in the absence of wood in the media. Many researchers have reported the same morphological changes in biodegraded LDPEs [37,40–42]. Damages to the LDPE may be due to the extracellular metabolites and enzymes released by the fungus or due to mechanical force applied by the fungus in response to stress. *Phlebiopsis flavidoalba* KH2 treated LDPE had the highest percentage weight loss and macroscopic peeling off of the polyethylene sheet was even evident to the naked eye. In unwashed LDPE sheets, mycelial mats were observed under the unaided eye as well as in the SEM images indicating that some kind of adherence or physical attachment is required for the degradation. Gómez-Méndez et al. (2018) [10] described that the formation of a biofilm on the polymer’s surface is required during microbial polymer degradation. Microorganisms colonizing the polymer could also have adhered to its surface through chitin and glycan production [43]. These biological polymers covalently adhere to the LDPE surface and play an important role in depolymerizing enzyme support and transport during the superficial attack [43]. Furthermore, there is a possibility to produce hydrophobins, self-aggregating proteins that form films, when exposed to hydrophobic-hydrophilic natural interphases [44].

The metabolic activities of microorganisms in culture media are evident in their pH changes as well as in their enzymatic activities. It has been reported that fungal-mediated wood decay generally creates an acidic environment [45]. Most interesting thing is even in the presence of LDPE as the sole C source, pH of the media shifted towards the basic direction (from 5.4 to 8.2) except for two fungal isolates. Further, lignolytic enzyme activities were also detected when the only C source is LDPE indicating the fungal activities and active metabolism. Lignolytic enzyme activities have been directly reported to be associated with both lignin and polyethylene degradation. For example, Fujisawa et al. [34] reported enhanced degradation of polyethylene membrane after treating with laccase from *Trametes versicolor* in the presence of 1-hydroxybenzotriazole as a mediator. Sowmya et al. [46] reported both LiP and MnP are essential for the degradation of lignin as well as for oxidizing polyethylene. Iiyoshi et al. [6] found MnP’s involvement in polyethylene degradation. In the present study, a relatively small but significant positive correlation was detected between MnP activity and the reduction in tensile properties of the LDPE sheets. Further, since CO_2_ released was confirmed by *P*. *flavidoalba* KH2 in LDPE-containing media indicating effective utilization and degradation, we obtained the full genome sequence of *P*. *flavidoalba* (assembled sequences deposited at NCBI under BioProject ID PRJNA879914 with BioSample ID SAMN30816873) and used in molecular docking (unpublished data). It was found that alkane having C 18 (Octadecane molecule) docked nicely with LiP with a binding affinity of -6.6 kcal/mol. Leu140, Pro111, Met139, His143, Phe11, ILE120, Ala142, Phe8, Phe231, Phe227, Leu7 and Leu234 amino acid residues of the modeled LiP interacted with Octadecane where alkyl bonds were present [unpublished data] further supporting the wet lab experiments.

Though it is clear that white rot fungi can attack polyethylene, the exact mechanism of degradation is not clear. However, C limited setting seems to induce the deterioration. Iiyoshi et al. [6] suggested that lignin-degrading fungi deteriorated the high-molecular-weight polyethylene even in the absence of supplied carbon (C) and nitrogen (N) sources. Santo et al. [38] demonstrated that the microbial cell surface becomes more adhesive in carbon-starved cultures than in non-starved cultures. This indicates that these fungi have metabolic flexibility to adjust and utilize available C sources for their survival. Further researchers have described that some chemical properties were similar between lignin and polyethylene and their degradation patterns as well. Lignin is a phenylpropanoid polymer having C-C and methyl ester bonds and similarly recalcitrant C-C bonds do occur in polyethylene. Both are hydrophobic in nature. Even though plastics/polyethylene have higher bond dissociation energies than that of lignin [47], it is proposed that abiotic factors like UV [48] and with both hydrolytic and oxidative enzyme activities facilitate both lignin as well as polyethylene degradation [7]. It is also reported that ligninolytic fungi generate a series of free radicals, such as peroxides, superoxides, and hydroxyls, which act as oxidation-diffusible mediators for polyethylene degradation before the enzyme attack [10,49]. One such method to generate these oxidative stress in ligninolytic fungi is via H_2_O_2_ generating enzymes as reported in Dashtban et al. [49]. Taking all the factors into consideration, here we propose a potential mechanism for white rot mediated LDPE degradation as follows.

The process of biodeterioration of LDPE and enzymatic activities was conceptualized by evaluating the overall results of the experiment particularly with FTIR analysis, and supporting literature. However, the exact enzymatic mechanisms and activities of fungi on LDPE is yet to be fully understood. At the beginning, fungi are attached to the LDPE surface, and they are glued on the surface. Fungal exopolysaccharides play a major role here as reported in other literature [43,50]. In the present study, it was evident that the mycelia were attached to the LDPE surface which induces the secretion of extracellular enzymes. Though there is no strong positive correlation between ligninoolytic enzyme production and deterioration, the correlation was significant. Weak correlation is a result of the fact that we used different fungal species that each works under different genetic and environmental backgrounds. Along with various other enzymes, MnP, LiP, and laccase might have played a major role as suggested by Ghatge et al. [51]. With the fungal enzyme-mediated photo-oxidation, the degradation process of the LDPE should be initiated by the formation of radicals that help in the oxidation of the polymer in the presence of light, oxygen, and water. Then the oxidized products converted to oxygen-containing functional groups through the Norrish type I and Norrish type II reactions [52]. In support of this, additional peaks were observed in the IR spectrum of the LDPE treated with the fungi due to the presence of vinylene group (−CH = CH−), and vinylene group with a terminal double bond (−CH = CH2). In the present study, peaks observed in FTIR for C-O or C = O stretching should be due to the formation of esters, ethers, as Norrish type I cleavage yields a carbonyl radical, which react with an alkoxy radical on the LDPE chain. It has been reported that hydrolytic enzymes such as cutinase, lipase, esterase, and alkane monooxygenase can mediate similar reactions with LDPE [53,54]. In the presence of dehydrogenase enzymes, the hydroxyl and ester intermediate formed during this process undergoes further oxidization and dehydrogenation, and smaller fatty acids are formed. During the metabolic process of beta-oxidation, these fatty acids undergo the formation of acetyl CoA, which is finally converted to carbon dioxide and water through the citric acid cycle [55].

## Conclusion

Here we report that fungi isolated from decaying hardwood, particularly from decay-resistant hardwood, are capable of deteriorating LDPE sheets. Deterioration was confirmed by direct or indirect means such as weight loss, reduction in tensile properties, reduction in hydrophobicity, changes in active groups in FTIR analysis, and SEM observations. In almost all the cases, signatures of deterioration were significant when the culture media did not contain any other C source. In other words, when the only C source was LDPE, an elevated level of deterioration was reported displaying the metabolic plasticity of fungi. Out of several fungal species showing signatures of deterioration, one fungal species, *Phlebiopsis flavidoalba*, isolated from a decay resistant native wood species, could utilize LDPE as the C source as evident by the Strum test. Further, deterioration and lignolytic enzyme activities were found to be correlated and particularly, LiP of *P*. *flavidoalba* showed binding affinity to octadecane, CH_3_(CH_2_)16CH_3_. Finally, potential mode of LDPE degradation via Norrish type I and Norrish type II reactions was also proposed.

## Supporting information

S1 File (ZIP)
