# Supplementary figures and images for: Wood decay fungi show enhanced biodeterioration of low-density polyethylene in the absence of wood in culture media
Source: PLoS One. 2023 Jul 26;18(7):e0288133. doi: 10.1371/journal.pone.0288133 (PMC10370761; doi:10.1371/journal.pone.0288133)

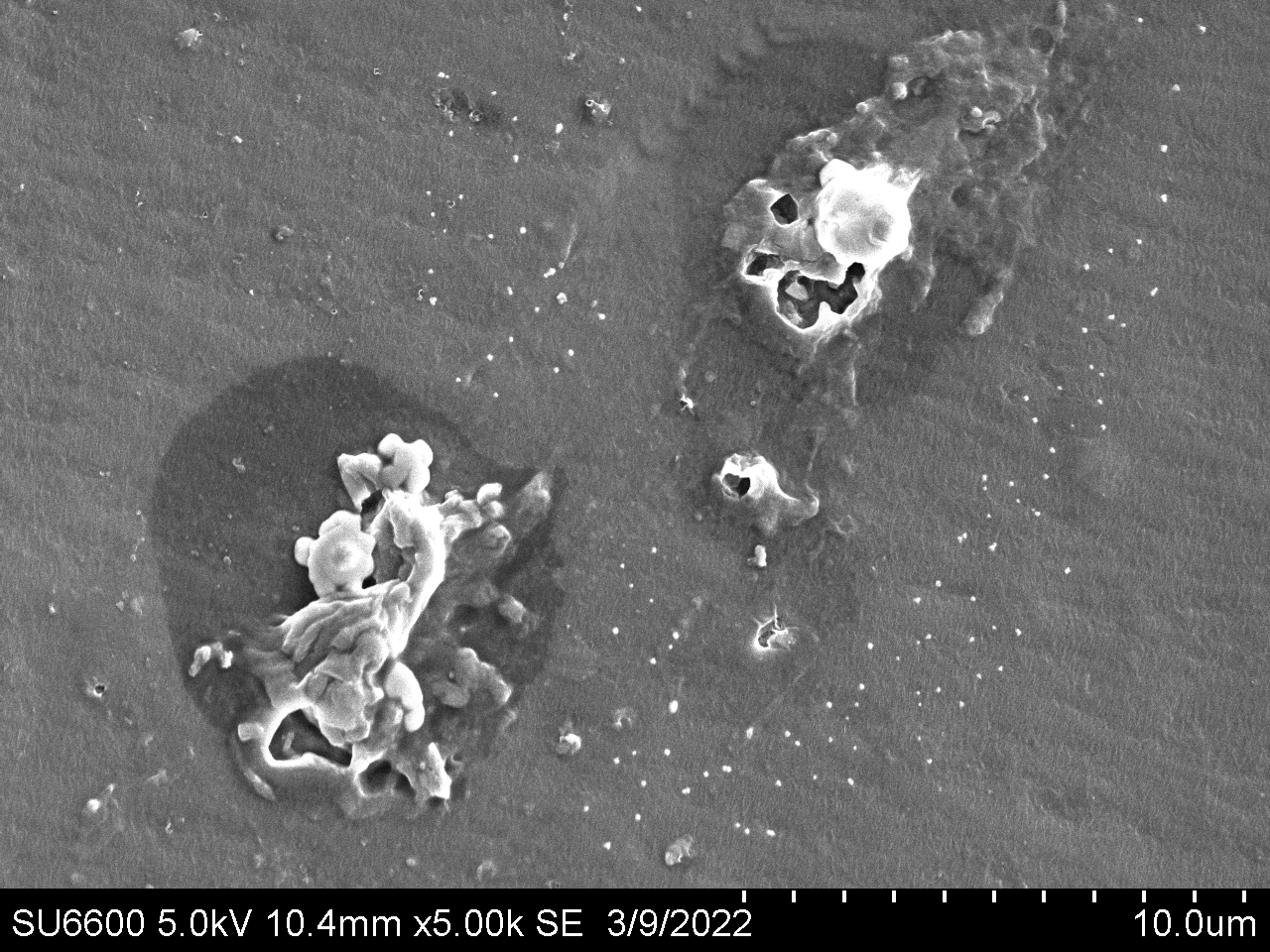

Supplement: S1 File — (ZIP) [file pone.0288133.s001.zip › SEM salt medium/DD18_m01.tif]

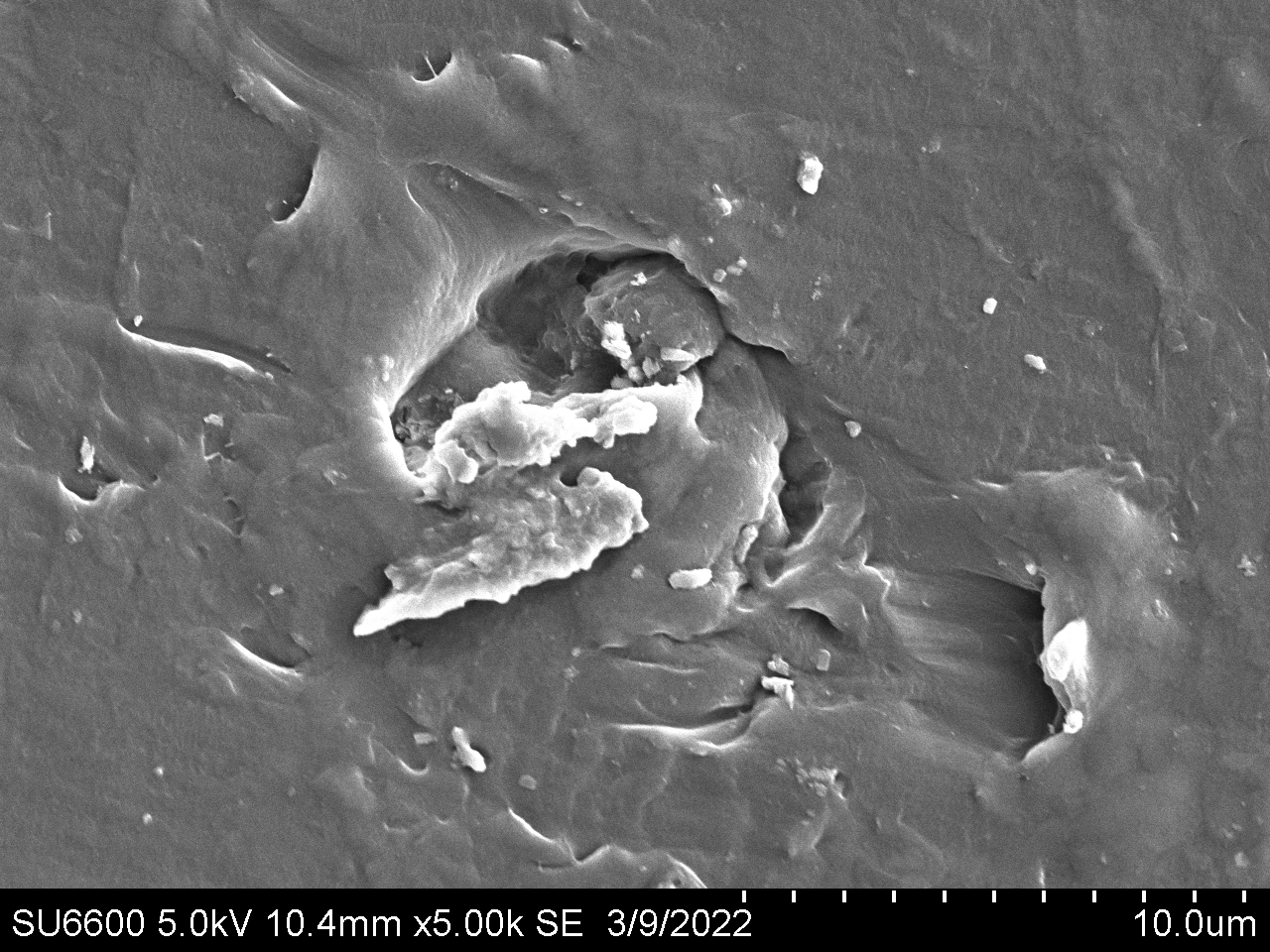

Supplement: S1 File — (ZIP) [file pone.0288133.s001.zip › SEM salt medium/DD18_m02.tif]

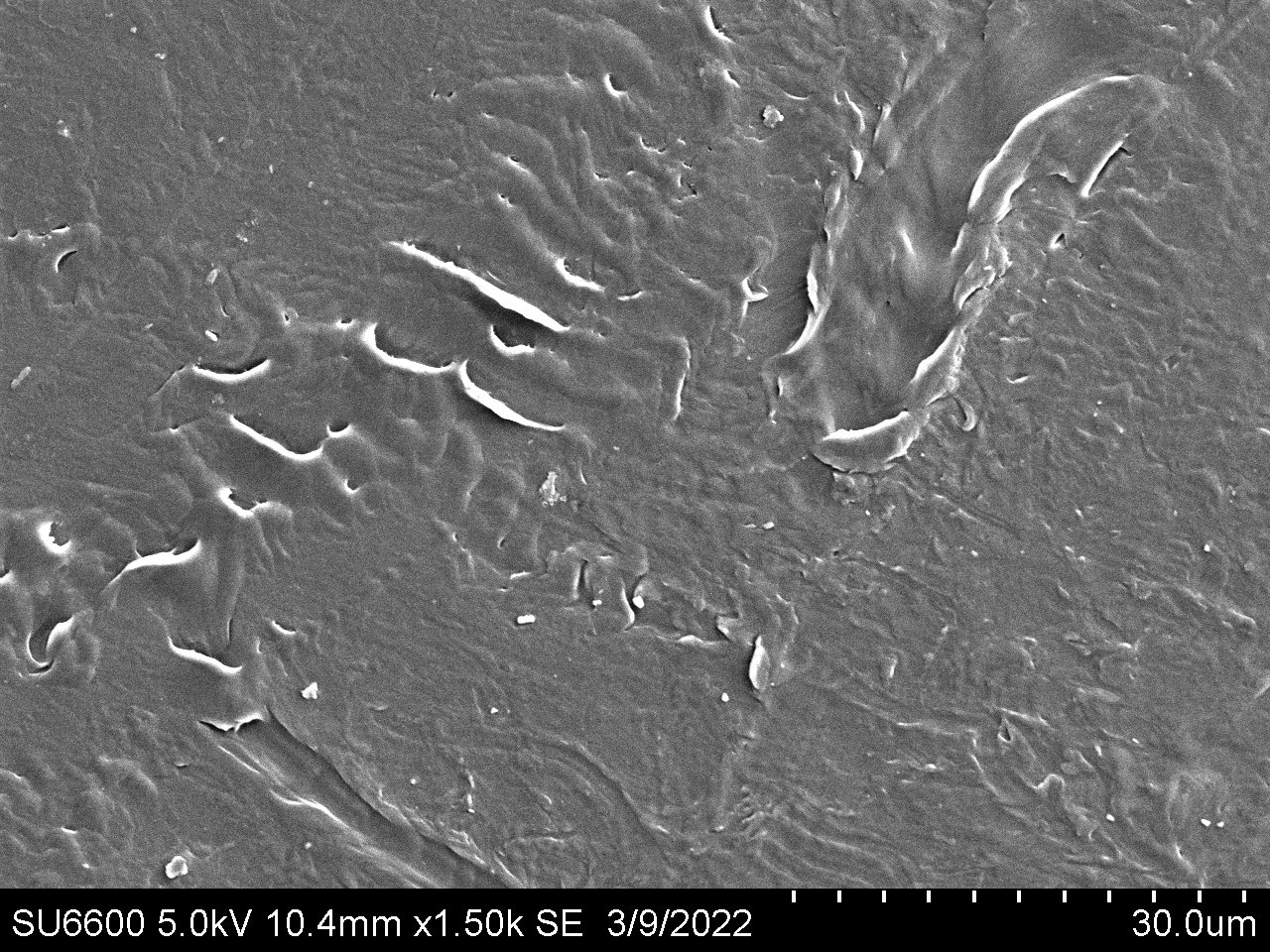

Supplement: S1 File — (ZIP) [file pone.0288133.s001.zip › SEM salt medium/DD18_m03.tif]

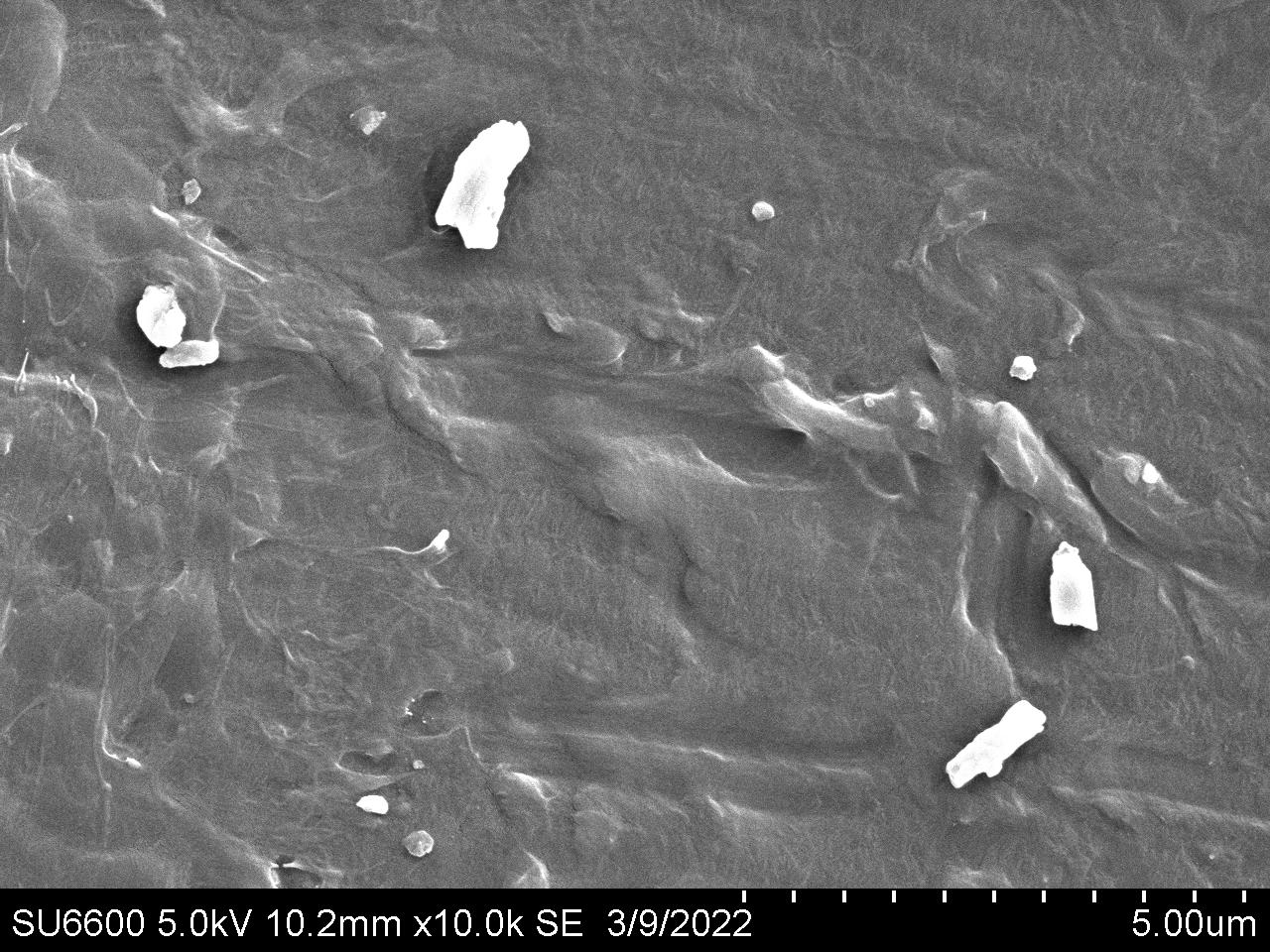

Supplement: S1 File — (ZIP) [file pone.0288133.s001.zip › SEM salt medium/DD18_m05.tif]

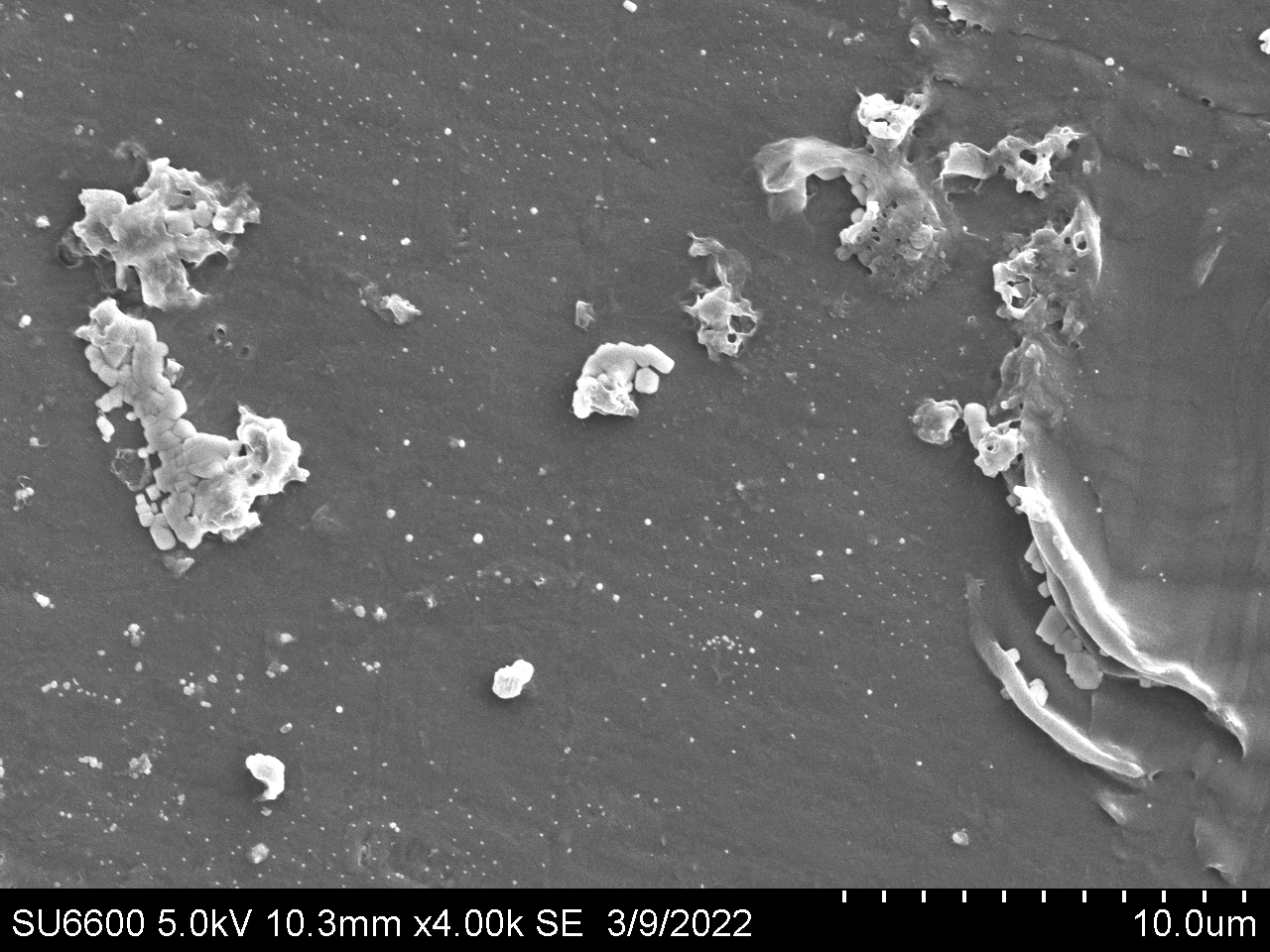

Supplement: S1 File — (ZIP) [file pone.0288133.s001.zip › SEM salt medium/DD18_m06.tif]

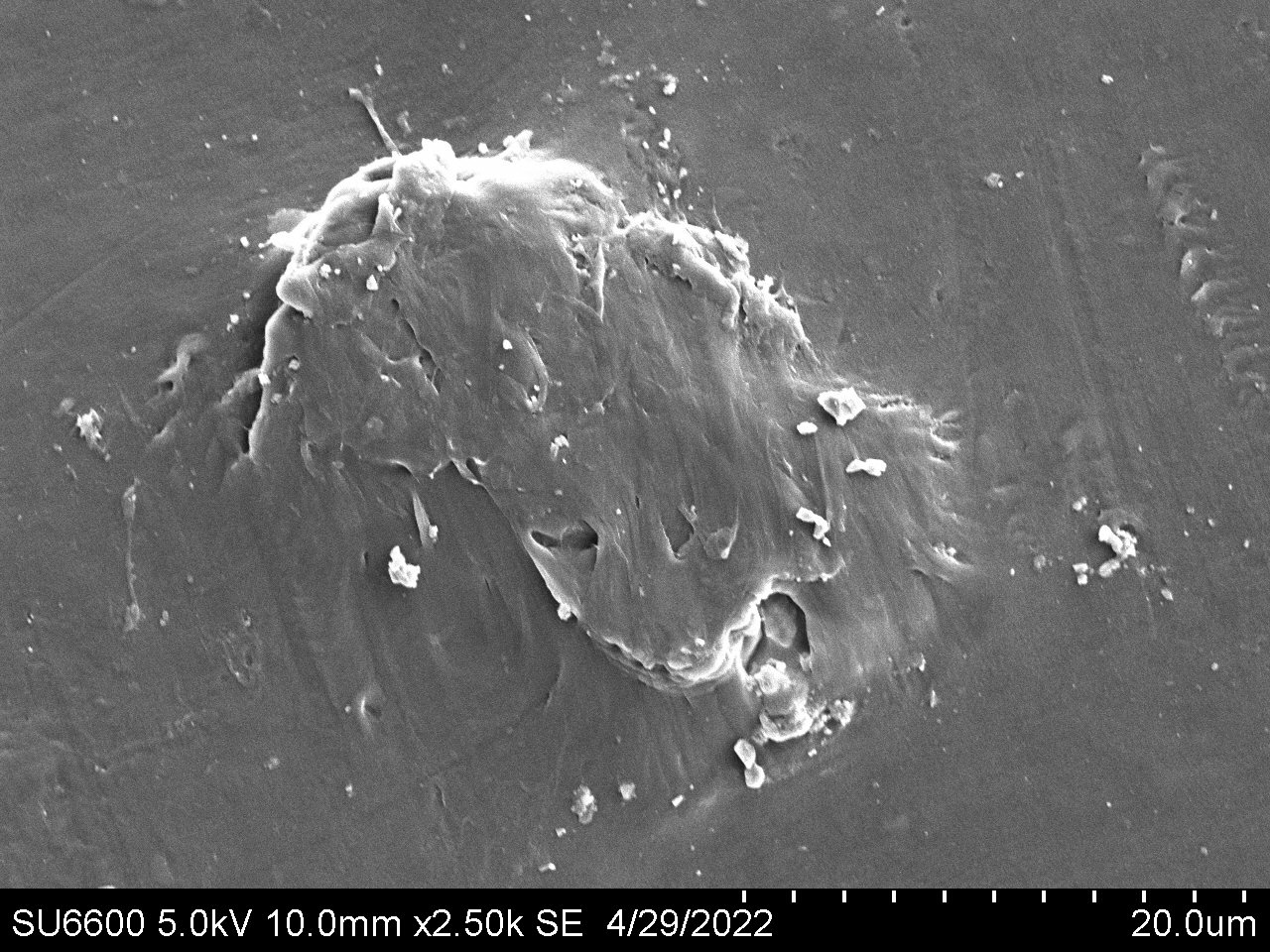

Supplement: S1 File — (ZIP) [file pone.0288133.s001.zip › SEM salt medium/DD27_m01.jpg]

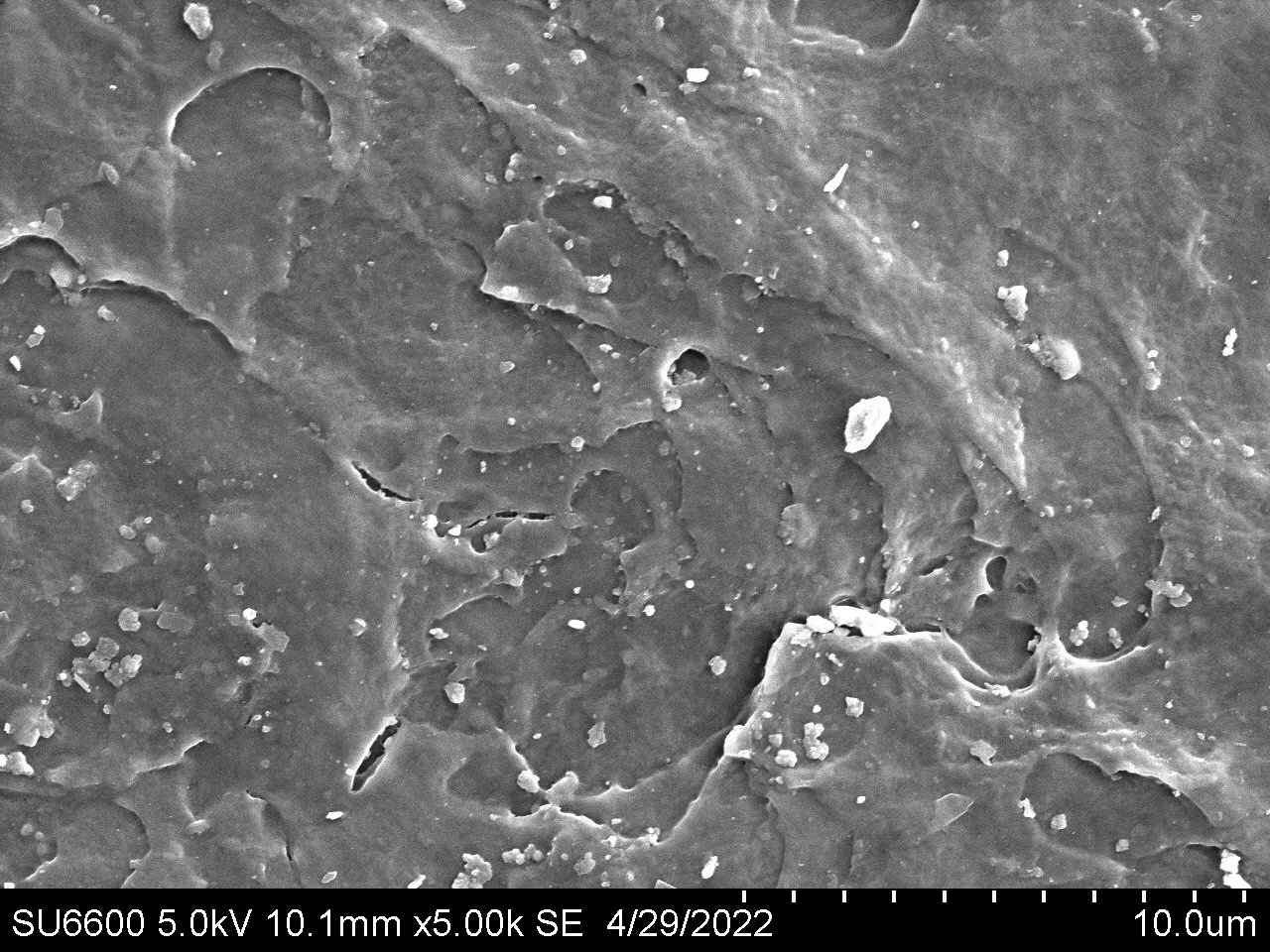

Supplement: S1 File — (ZIP) [file pone.0288133.s001.zip › SEM salt medium/DD27_m02.jpg]

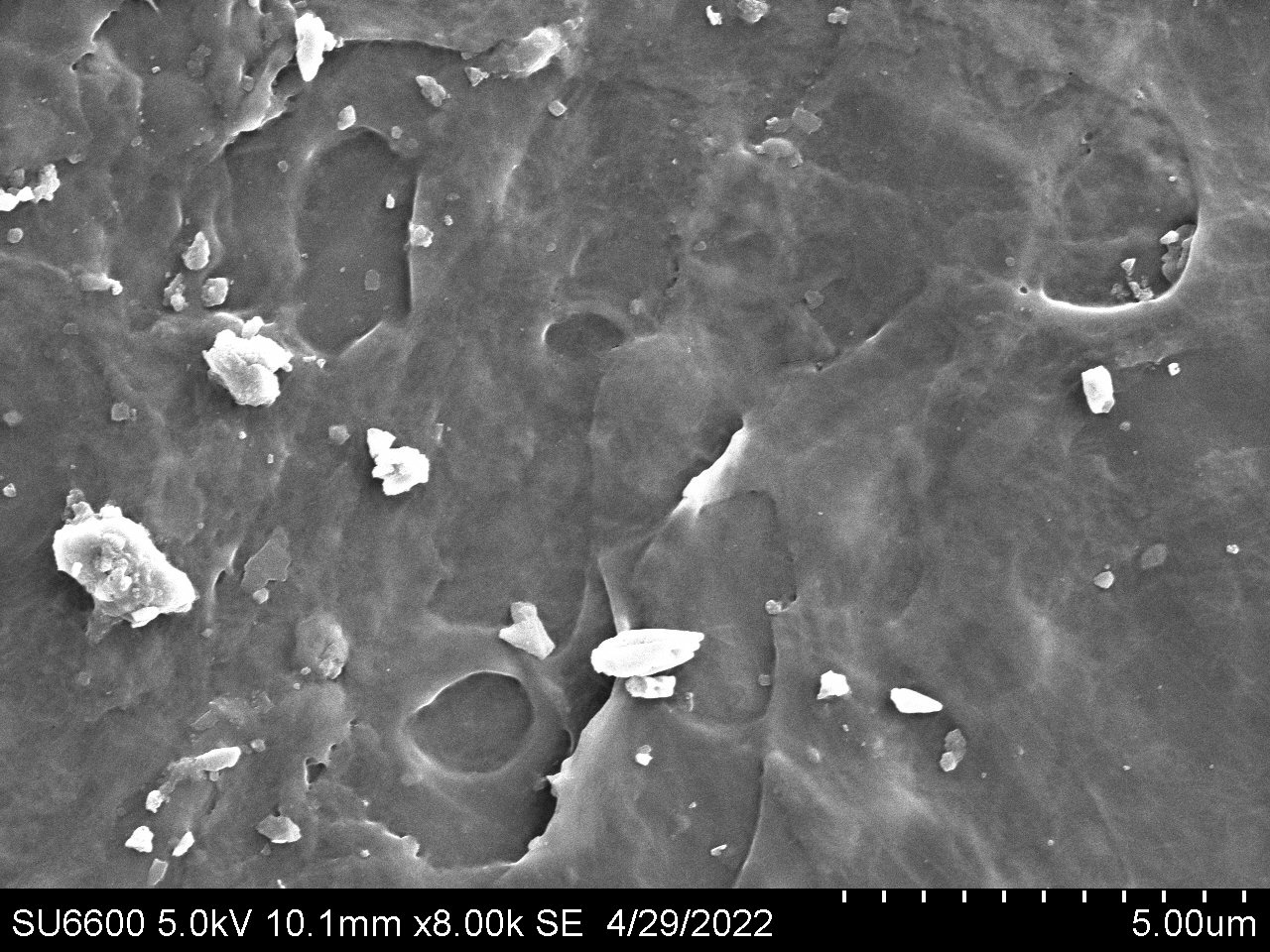

Supplement: S1 File — (ZIP) [file pone.0288133.s001.zip › SEM salt medium/DD27_m03.jpg]

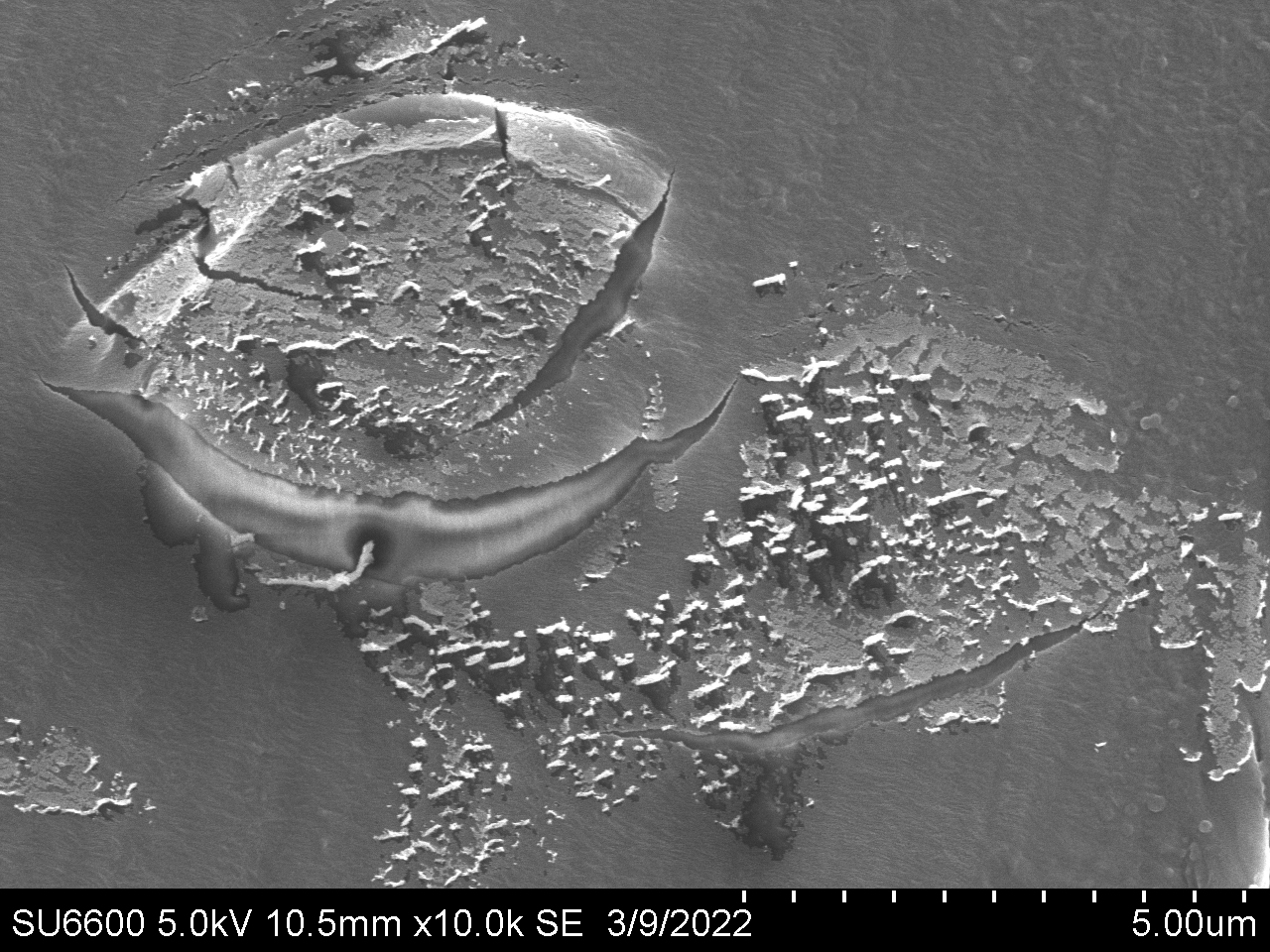

Supplement: S1 File — (ZIP) [file pone.0288133.s001.zip › SEM salt medium/DD28_m01.tif]

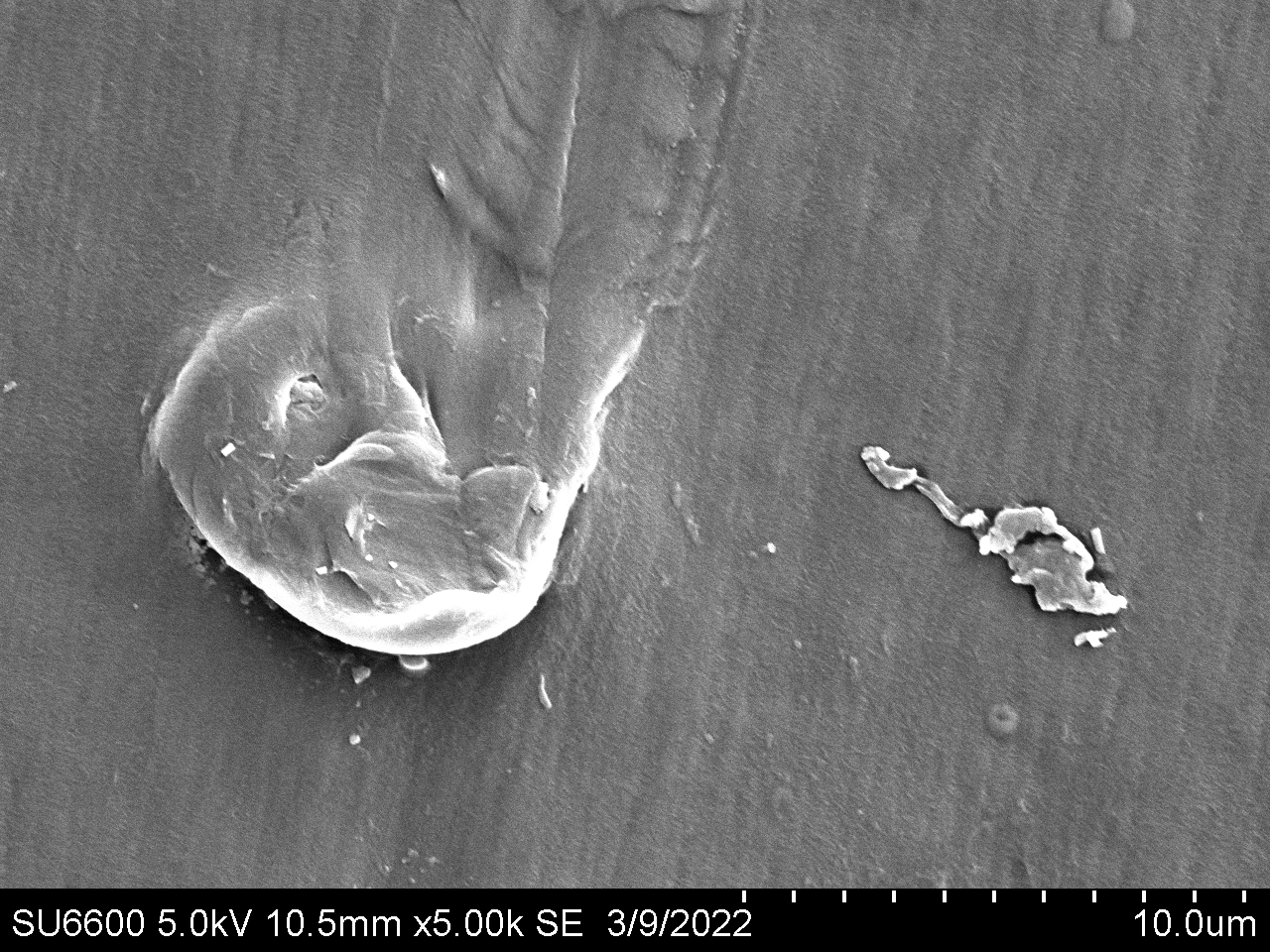

Supplement: S1 File — (ZIP) [file pone.0288133.s001.zip › SEM salt medium/DD28_m03.tif]

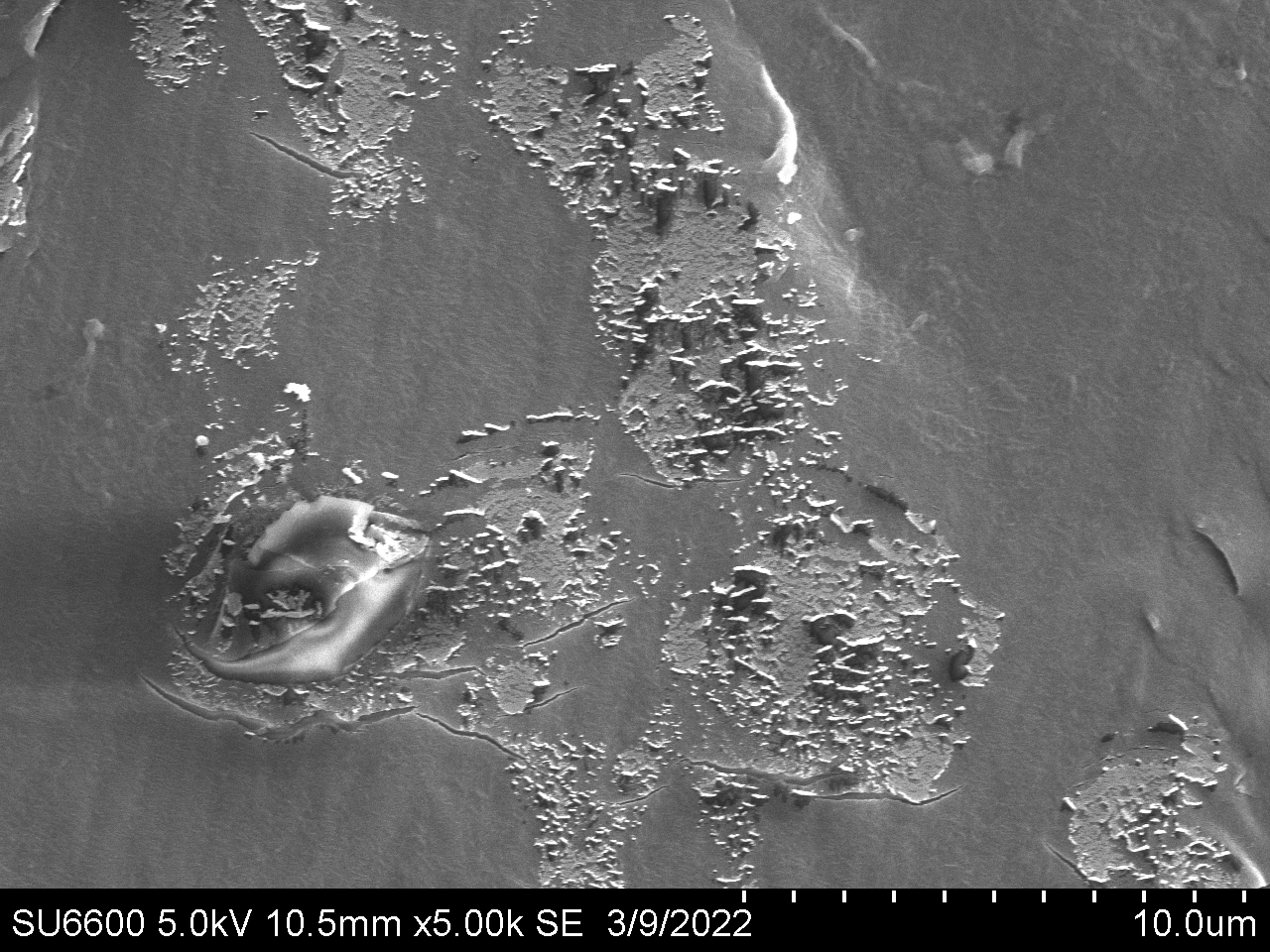

Supplement: S1 File — (ZIP) [file pone.0288133.s001.zip › SEM salt medium/DD28_m06.tif]

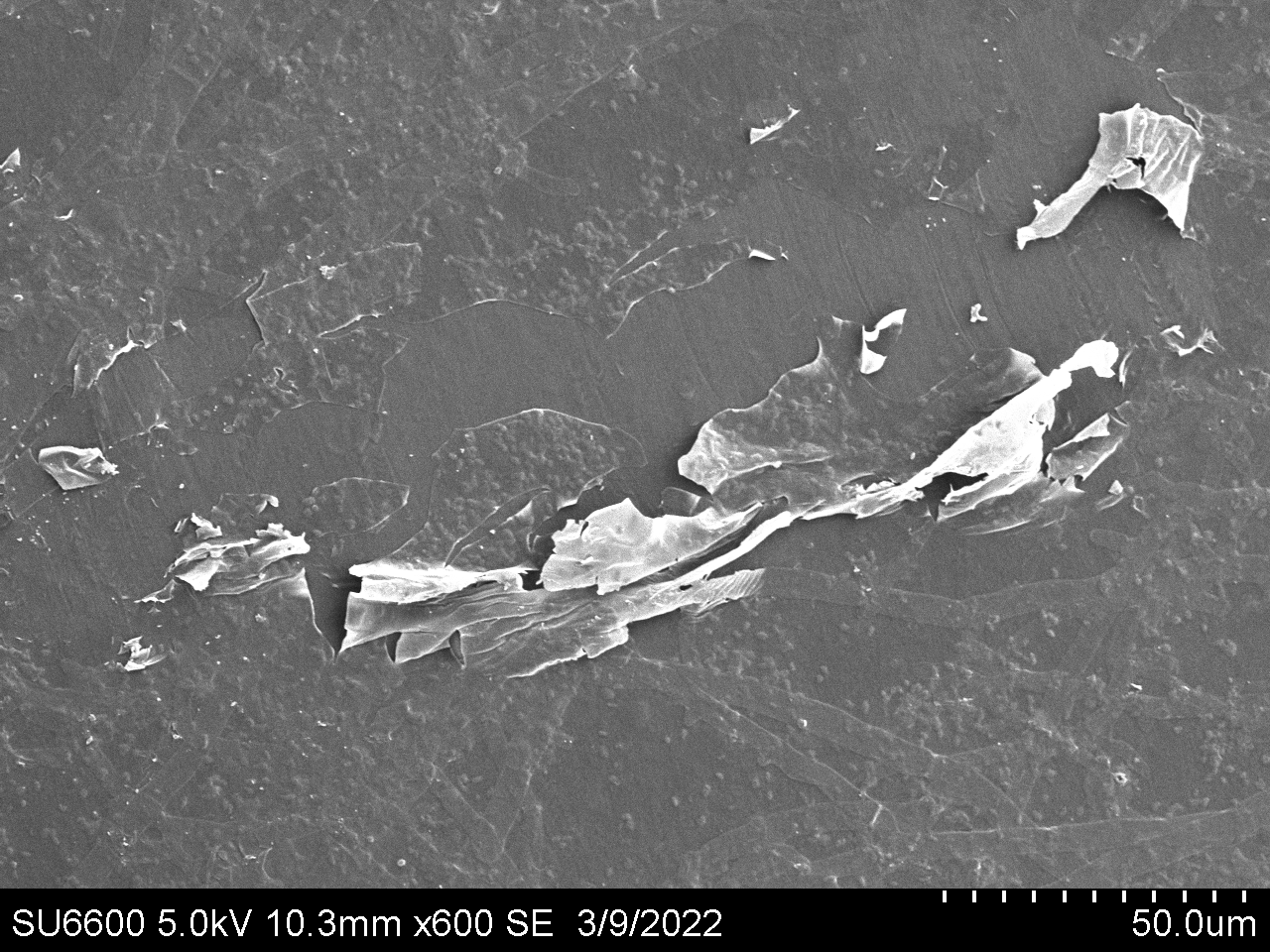

Supplement: S1 File — (ZIP) [file pone.0288133.s001.zip › SEM salt medium/DD31_m01.tif]

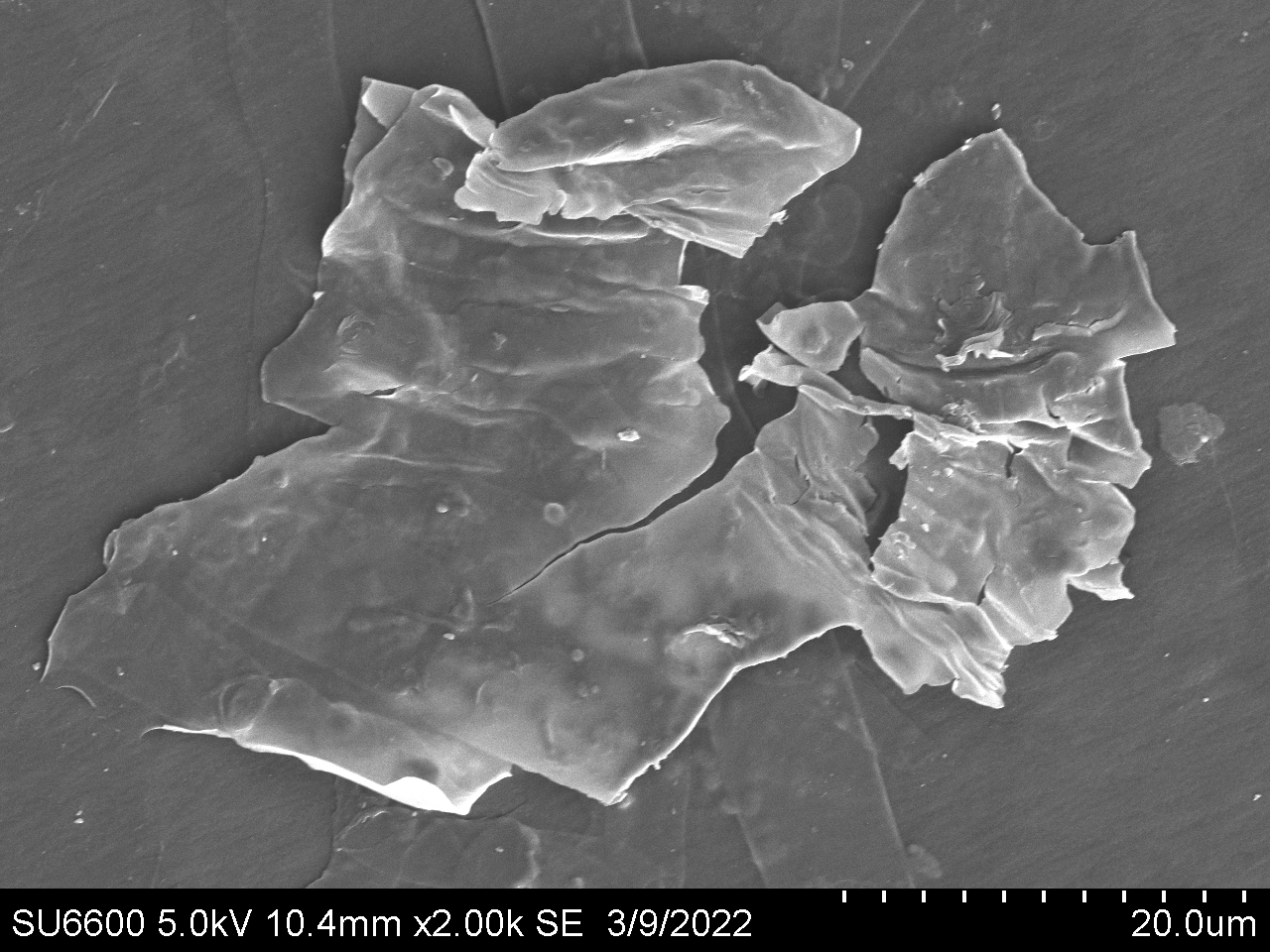

Supplement: S1 File — (ZIP) [file pone.0288133.s001.zip › SEM salt medium/DD31_m03.tif]

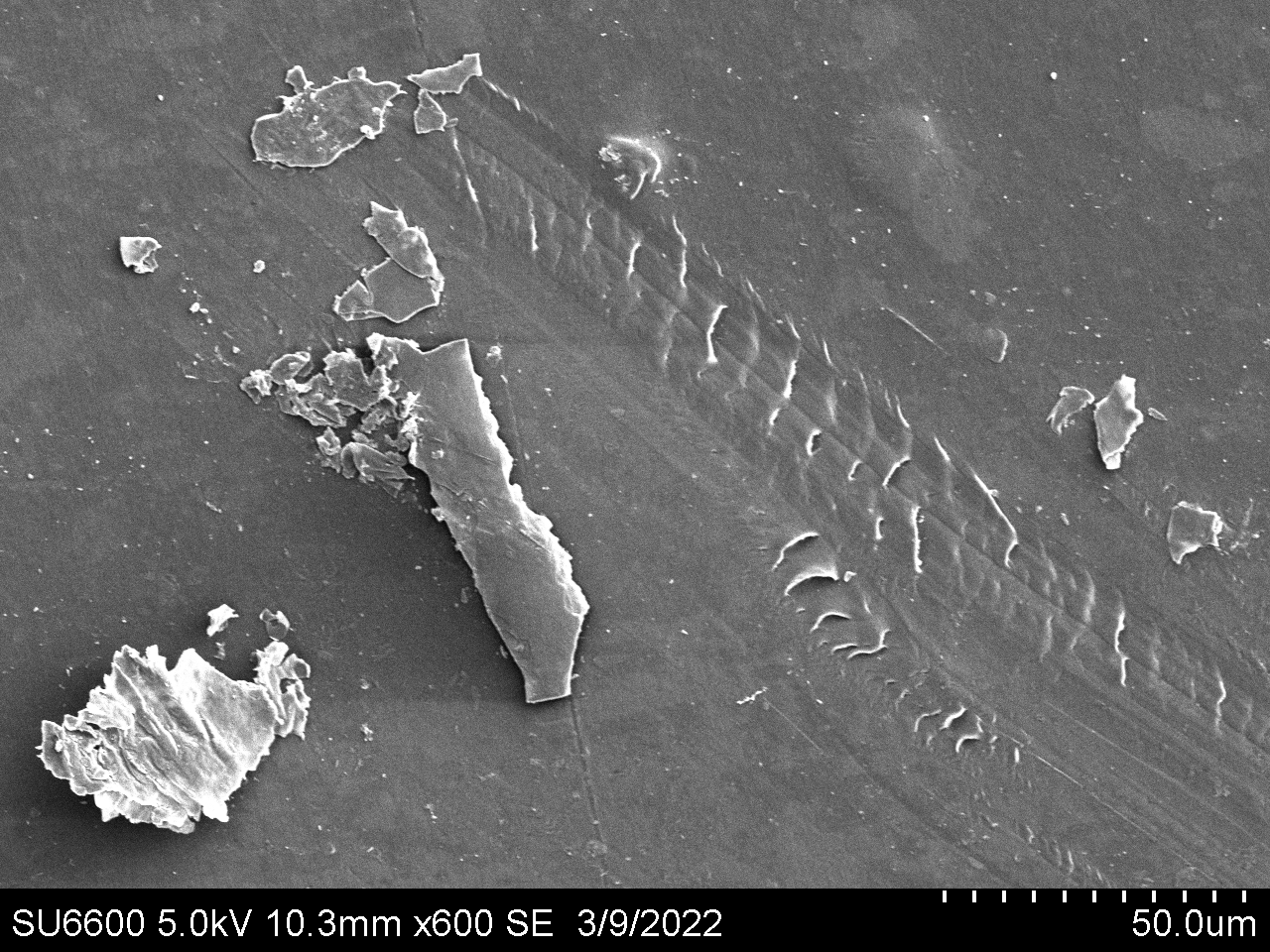

Supplement: S1 File — (ZIP) [file pone.0288133.s001.zip › SEM salt medium/DD31_m06.tif]

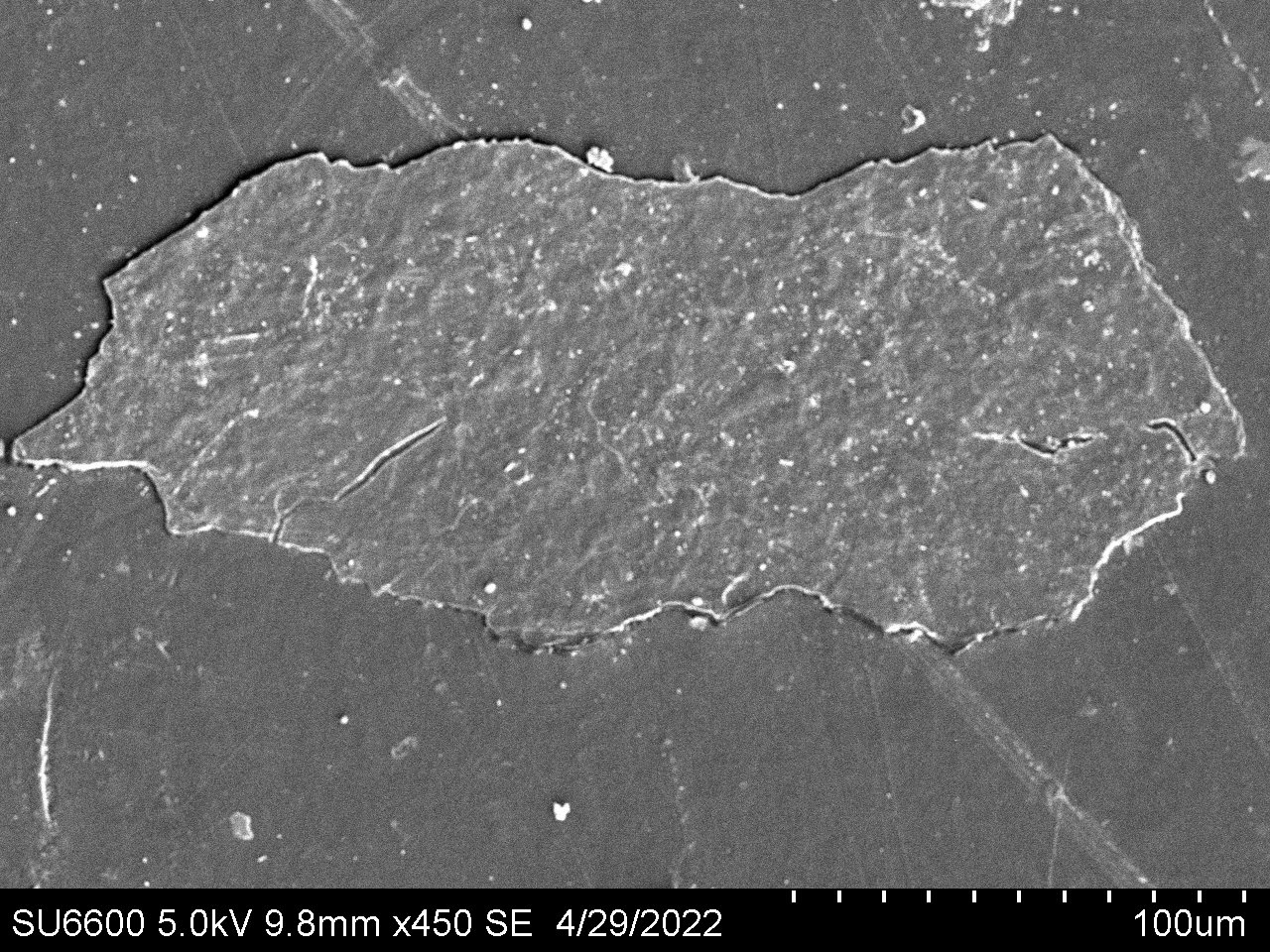

Supplement: S1 File — (ZIP) [file pone.0288133.s001.zip › SEM salt medium/KH2_m05.jpg]

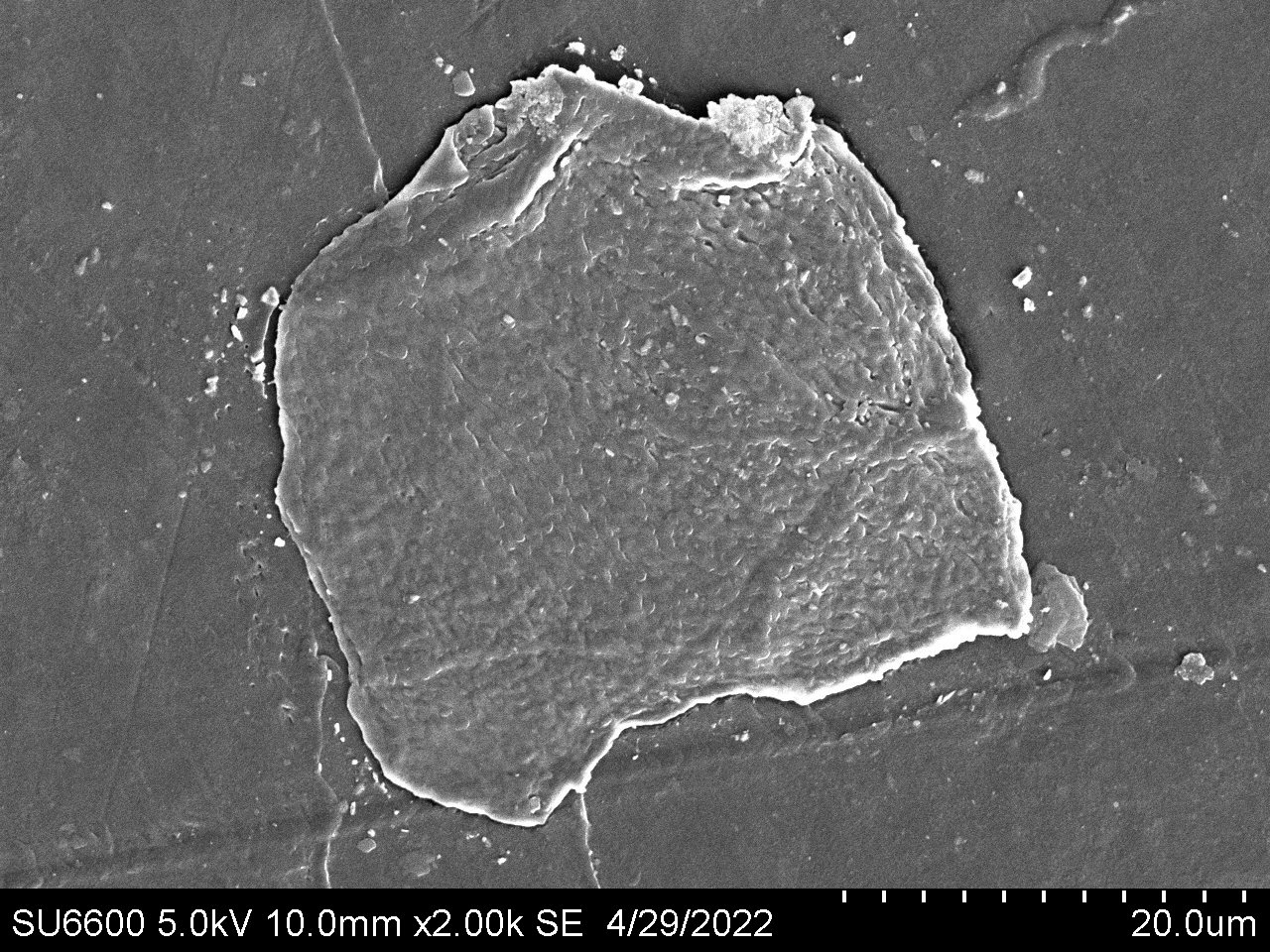

Supplement: S1 File — (ZIP) [file pone.0288133.s001.zip › SEM salt medium/KH2_m06.jpg]

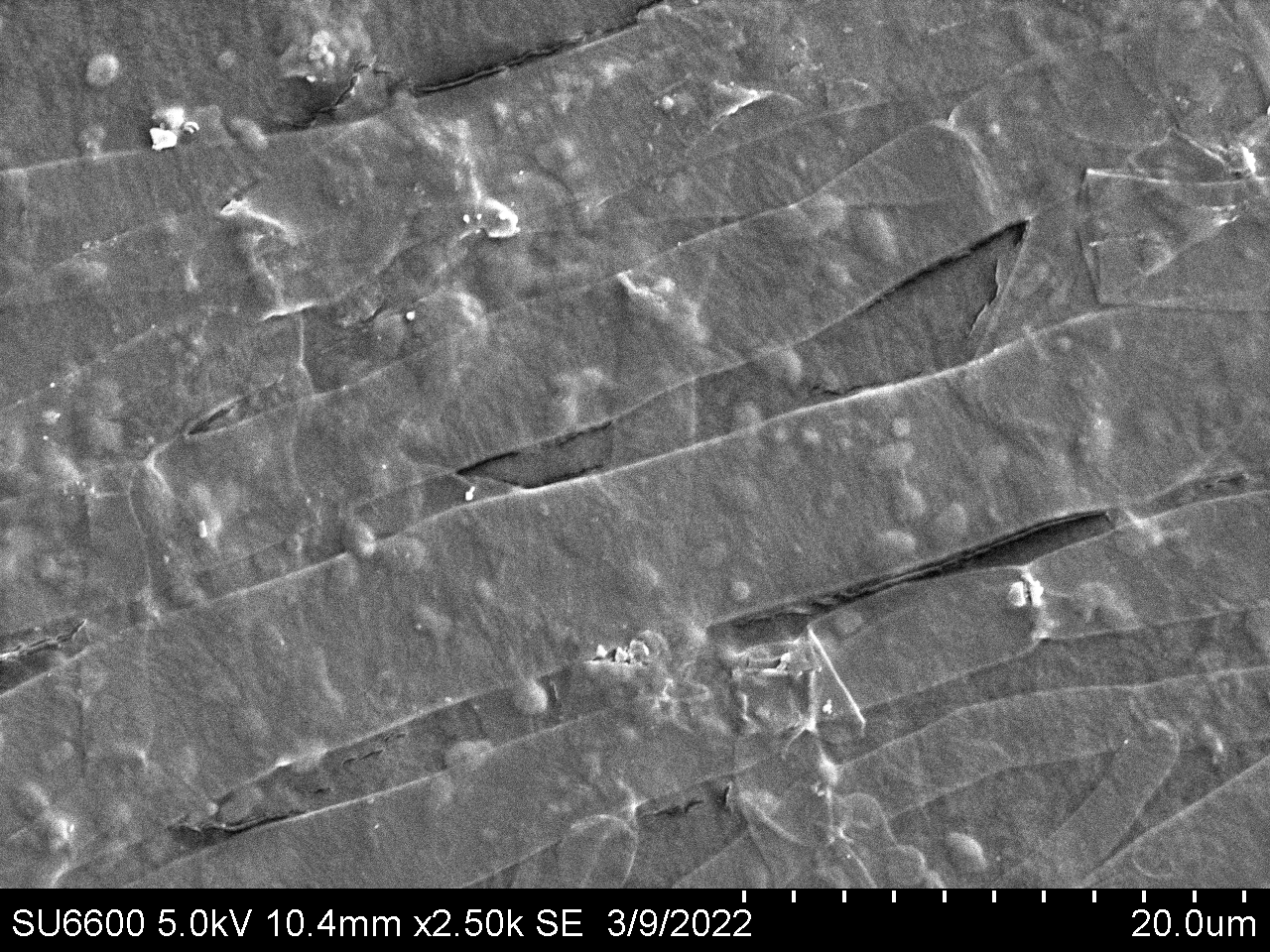

Supplement: S1 File — (ZIP) [file pone.0288133.s001.zip › SEM salt medium/unwashed DD31_m05.tif]
